# New Alkoxy Flavone Derivatives Targeting Caspases: Synthesis and Antitumor Activity Evaluation

**DOI:** 10.3390/molecules24010129

**Published:** 2018-12-31

**Authors:** Joana Moreira, Diana Ribeiro, Patrícia M. A. Silva, Nair Nazareth, Madalena Monteiro, Andreia Palmeira, Lucília Saraiva, Madalena Pinto, Hassan Bousbaa, Honorina Cidade

**Affiliations:** 1 Laboratory of Organic and Pharmaceutical Chemistry, Department of Chemical Sciences, Faculty of Pharmacy, University of Porto, Rua Jorge Viterbo Ferreira, 228, 4050-313 Porto, Portugal; joana.m26@hotmail.com (J.M.); andreiapalmeira@gmail.com (A.P.); madalena@ff.up.pt (M.P.); 2 Interdisciplinary Centre of Marine and Environmental Research (CIIMAR), University of Porto, Terminal de Cruzeiros do Porto de Leixões, Av. General Norton de Matos s/n, 4450-208 Matosinhos, Portugal; 3 CESPU, Institute of Research and Advanced Training in Health Sciences and Technologies (IINFACTS), Rua Central de Gandra, 1317, 4585-116 Gandra, Portugal; diana.ribeiro@cespu.pt (D.R.); patricia.silva@cespu.pt (P.M.A.S.); 4 LAQV/REQUIMTE, Laboratory of Microbiology, Department of Biological Sciences, Faculty of Pharmacy, University of Porto, Rua Jorge Viterbo Ferreira, 228, 4050-313 Porto, Portugal; naircampos@gmail.com (N.N.); madsmonteiro@gmail.com (M.M.); lucilia.saraiva@ff.up.pt (L.S.)

**Keywords:** flavonoids, *O*-heterocycles, alkylation, antitumor activity, apoptosis, caspase activators

## Abstract

The antitumor activity of natural flavonoids has been exhaustively reported. Previously it has been demonstrated that prenylation of flavonoids allows the discovery of new compounds with improved antitumor activity through the activation of caspase-7 activity. The synthesis of twenty-five flavonoids (**4**–**28**) with one or more alkyl side chains was carried out. The synthetic approach was based on the reaction with alkyl halide in alkaline medium by microwave (MW) irradiation. The in vitro cell growth inhibitory activity of synthesized compounds was investigated in three human tumor cell lines. Among the tested compounds, derivatives **6**, **7**, **9**, **11**, **13**, **15**, **17,** and **18** revealed potent growth inhibitory activity (GI_50_ < 10 μM), being the growth inhibitory effect of compound **13** related with a pronounced caspase-7 activation on MCF-7 breast cancer cells and yeasts expressing human caspase-7. A quantitative structure-activity relationship (QSAR) model predicted that hydrophilicity, pattern of ring substitution/shape, and presence of partial negative charged atoms were the descriptors implied in the growth inhibitory effect of synthesized compounds. Docking studies on procaspase-7 allowed predicting the binding of compound **13** to the allosteric site of procaspase-7.

## 1. Introduction

Caspases are a family of proteases with a crucial role in the initiation and execution of apoptosis [1]. At the core of the execution phase of apoptosis are the executioner caspases 3 and 7. These proteases are stored as procaspases, which, after activation by proteolysis, cleave a large set of substrates leading to apoptosis. Therefore, the search for activators of these procaspases has been attracting attention of the scientific community in the field of anticancer drug discovery, being demonstrated their potential as antitumor agents [2]. To date, although several compounds have proved to interfere with the caspase activity, most of them act on the signaling pathway involved in caspase activation, while few compounds have demonstrated directly interfering with caspases activity or allosteric sites.

Flavones are a class of oxygenated heterocyclic natural products with a benzo-γ-pyrone scaffold that have long been recognized for their potent antitumor activity, associated, at least in part, with their ability to induce apoptosis [3]. Baicalein (**1**), 3,7-dihydroxyflavone (**2**) and chrysin (**3**) have been reported as antitumor agents, by acting as inducers of apoptosis in human tumor cell lines through caspases-dependent pathways [4,5,6,7]. Our research group previously reported that the introduction of prenyl side chains on flavonoids scaffolds, including baicalein (**1**) and 3,7-dihydroxyflavone (**2**), was associated with an increase in their growth inhibitory activities towards several human tumor cell lines [8,9,10,11], being this effect related to the activation of caspase-7 for some of the prenylated derivatives [12]. Moreover, some studies have demonstrated that the alkylation of flavone scaffold was associated with an improvement of antitumor activity [13,14]. In fact, Wang et al. reported that the 7-*O*-alkylation of baicalein (**1**) and chrysin (**3**) with lipophilic alkyl groups results in the identification of some derivatives with stronger growth inhibitory effect in human colon cancer SW480 cells compared with the non-alkylated precursor [14].

Although natural hydroxyflavonoids have been shown to exert beneficial effects in in vitro assays, their low bioavailability has limited their success in in vivo experiments [15]. This could be due to the presence of hydroxyl groups, which are susceptible to rapid intestinal/hepatic conjugation by glucuronidation or sulfation [16]. In contrast, methylated hydroxyflavones, in comparison with unmethylated flavones, are reported to be relatively stable, indicating high resistance to hepatic metabolism, suggesting that the replacement of hydroxyl by methoxy groups could overcome the low bioavailability and poor metabolic stability of the compounds, increasing their potential for therapeutic application [17].

Taking these into account, we decided to synthesize and evaluate a series of baicalein (**1**), 3,7-dihydroxyflavone (**2**) and chrysin (**3**) alkyl derivatives possessing 1–5 carbon atoms, allowing the maintenance of the proper balance between hydrophilicity and lipophilicity essential to be a successful drug. Twenty-five alkoxy flavone derivatives (**4**–**28**) were synthesized and evaluated for their growth inhibitory effect on human tumor cell lines. A QSAR model to predict the growth inhibitory effect was also developed. For the most potent growth inhibitors, assays were performed aiming to evaluate their effect on apoptosis and caspases activation. To further understand the binding mode of the hit compound (**13**) in procaspase-7, docking studies on procaspase-7 binding pocket were performed.

## 2. Results and Discussion

### 2.1. Synthesis

**Scheme 1:**
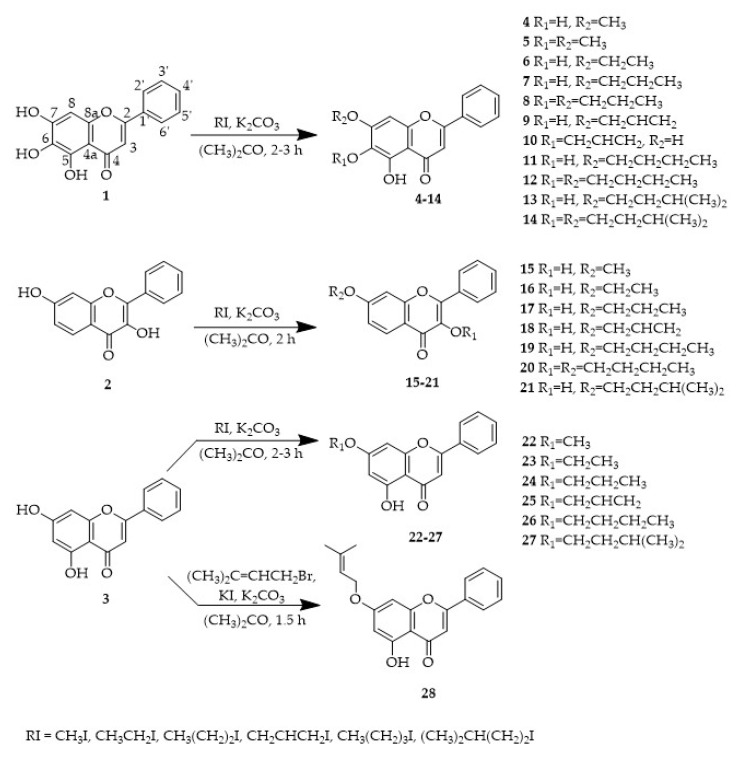
Synthesis of flavonoids **4**–**28**. The numbering used concerns the NMR assignments.

Twenty-five alkylated flavonoids were synthesized using baicalein (**1**), 3,7-dihydroxyflavone (**2**) and chrysin (**3**) as building blocks, according to the strategy illustrated in Scheme 1. The synthetic approach was based on the reaction of the building block with the alkyl halide, in presence of anhydrous potassium carbonate by MW irradiation. Among these compounds, eleven (**6**, **7, 13**, **14**, **17**–**21**, **24** and **27**) are here described for the first time.

For almost all alkylation reactions performed using baicalein (**1**) as building block, two alkylated derivatives were obtained. The major products were those with one alkyl side chain on the oxygen at position 7 (**4**, **6**, **7**, **9**, **11** and **13**), being the products with two alkoxy side chains on C-6 and C-7 (**5**, **8**, **12** and **14**) obtained as by-products. However, the reaction of baicalein (**1**) with allyl iodide produced a mixture of two monoalkylated derivatives **9** and **10**, being once again the derivative with the alkoxy side chain at position 7 (**9**) the major product. These results indicate that the hydroxyl group at C-7 is the first hydroxyl group to be alkylated.

For 3,7-dihydroxyflavone (**2**) and chrysin (**3**), only the monoalkylated derivatives **15**–**19** and **21**–**28** were isolated, except for the reaction of **2** with butyl iodide, for which the dialkylated derivative (**20**) was also obtained.

### 2.2. Structure Elucidation

The structure of new alkyl derivatives **6**, **7**, **13**, **14**, **17**–**21**, **24**, and **27** was established by infrared (IR), ^1^H and ^13^C nuclear magnetic resonance (NMR) (Figures S1–S11), and high-resolution mass spectrometry (HRMS) techniques (Figures S12–S22). The ^13^C NMR assignments were determined by bidimensional heteronuclear single quantum correlation (HSQC) and heteronuclear multiple bond correlation (HMBC) experiments. The structure elucidation of compounds **4**, **5**, **8**–**12**, **15**, **16**, **22**, **23**, **25**, **26** and **28** was established by comparing their IR, and ^1^H and ^13^C NMR data with those reported in the literature [13,14,18,19,20,21,22,23,24,25,26].

For instance, ^1^H and ^13^C NMR of compound **13** indicated the presence of a 5,6-dioxygenated flavone moiety and one isopentyloxy side chain. The assignments of the carbon atoms directly bonded to proton atoms were achieved from HSQC experiments, and the chemical shifts of the carbon atoms not directly bonded to proton atoms were deduced from HMBC correlations. The position of the isopentyloxy side chain was evidenced by the correlation found in the HMBC spectrum between the proton signals of H-1′′ (δ_H_ 4.18 t, *J* = 6.6 Hz) and the carbon signal of C-7 (δ_H_ 152.3).

### 2.3. Biological Activity

#### Growth Inhibitory Activity in Human Tumor Cell Lines

In previous studies, baicalein (**1**), 3,7-dihydroxyflavone (**2**) and chrysin (**3**), as well as the 7-prenyl derivatives of **1** and **2** were already shown to have in vitro cell growth inhibitory activity in human tumor cell lines [10,27]. Compounds **1** and **2** presented a concentration required to reduce growth rates to 50% (GI_50_) in A375-C5 (IL-1 insensitive malignant melanoma), MCF-7 (breast adenocarcinoma), and NCI-H460 (non-small-cell lung cancer) of 9–14 µM and 10–15 µM, respectively [10]. Compound **3** exhibited a GI_50_ value of 19.5 µM in MCF-7 cell lines [27]. When comparing the GI_50_ values obtained for baicalein (**1**) and 3,7-dihydroxyflavone (**2**) with those obtained for their 7-prenyl derivatives, it was demonstrated that the presence of a prenyl group on position 7 of baicalein (**1**) was associated with an increase on the growth inhibitory activity (GI_50_ values between 4.7 and 8.7 µM), while the 7-prenylated derivative of **2** exhibited a growth inhibitory activity similar to 3,7-dihydroxyflavone (**2**) towards the three human tumor cell lines (GI_50_ values between 12.5 and 17.0 µM) [10]. In the present study, the cell growth inhibitory activities of alkyl derivatives **4**–**28** were evaluated for their in vitro growth inhibitory effect on the same human tumor cell lines (Table 1).

Results are presented as the concentrations that were able to cause 50% cell growth inhibition (GI_50_) after a continuous exposure of 48 h and represent means ± SEM from at least three independent experiments performed in duplicate. Doxorubicin (positive control): A375, GI_50_ = 15.25 ± 1.20 nm; MCF-7, GI_50_ = 3.21 ± 1.37 nm; and NCI-H460, GI_50_ = 7.37 ± 1.78 nM.

Among all tested compounds, 7-monoalkylated derivatives **6**, **7**, **9**, **11**, **13**, **15**, **17** and **18** demonstrated the best results of GI_50_ (3.17–10.78 μM) in the three human tumor cell lines studied, pointing them as promising agents for further antitumor studies. The comparison of the GI_50_ values of these compounds with those previously described for baicalein (**1**) and 3,7-dihydroxyflavone (**2**) [10] used as building blocks demonstrates that the introduction of a 7-alkyl side chain is associated with an increase on the growth inhibitory activity. Nevertheless, for 7-alkylated chrysin (**3**) derivatives **22**–**28**, the alkylation failed to give potent growth inhibitors. Moreover, baicalein (**1**) derivatives **9**, **11** and **13** and 3,7-dihydroxyflavone (**2**) derivatives **15**, **17** and **18** revealed lower GI_50_ values than those published before for 7-prenylated derivatives of both building blocks [10], reinforcing the importance of this molecular modification to improve the growth inhibitory effect.

**Figure 1:**
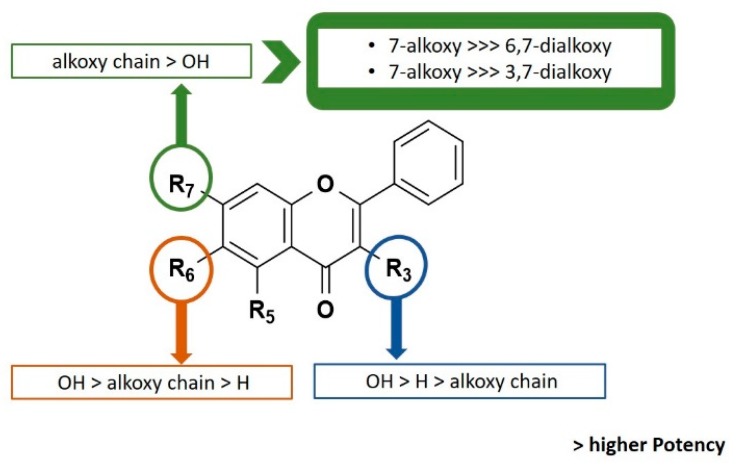
Structure-activity relationship for growth inhibitory activity in human tumor cell lines.

Interestingly, the number and position of alkoxy and hydroxyl groups in the flavone scaffold seems to influence the growth inhibitory effect (Figure 1). In fact, by comparing the structures **7**, **11**, **13**, and **19** with **8**, **12**, **14**, and **20**, respectively, it can be concluded that the presence of more than one alkoxy side chain on the flavone scaffold was associated with a reduction or complete loss of activity up to the concentrations tested (GI_50_ > 150 μM). Nevertheless, while the monomethylated baicalein derivative **4** showed to be inactive at the highest concentration tested (150 μM), derivative **5** with two methyl groups showed some activity. Furthermore, the results obtained for monoalkylated baicalein derivatives **9** and **10** suggested that the presence of an allyl side chain at C-7 is more favorable than at C-6. It is worth mentioning that these results are in accordance with the results previously reported by us for baicalein (**1**) and chrysin (**2**) prenylated derivatives [10]. Actually, the better antiproliferative activity of flavones with one alkyl side chain at C-7 here reported are quite similar to those obtained before for baicalein and 3,7-dihydroxyflavone prenylated derivatives [10].

On the other hand, the number and position of hydroxyl groups seems to also be important for the antiproliferative effect. When comparing the results obtained for monoalkylated baicalein derivatives **6**, **7**, **9**, **11**, **13** with chrysin derivatives **22**–**27** possessing the same alkoxy side chain, it appears that the presence of a 6-hydroxyl group at baicalein derivatives is associated with an improvement of activity. Furthermore, the presence of a hydroxyl group at C-3 on 3,7-dihydroxyflavone derivatives seems to be more favorable for activity than the presence of the same substituent at C-5. Actually, the GI_50_ values obtained for chrysin derivatives **22**–**27** are higher from those obtained for 3,7-dihydroxyflavone derivatives (**15**–**19**, and **21**), which might suggest the importance of the hydroxyl group on position 3.

**Figure 2:**
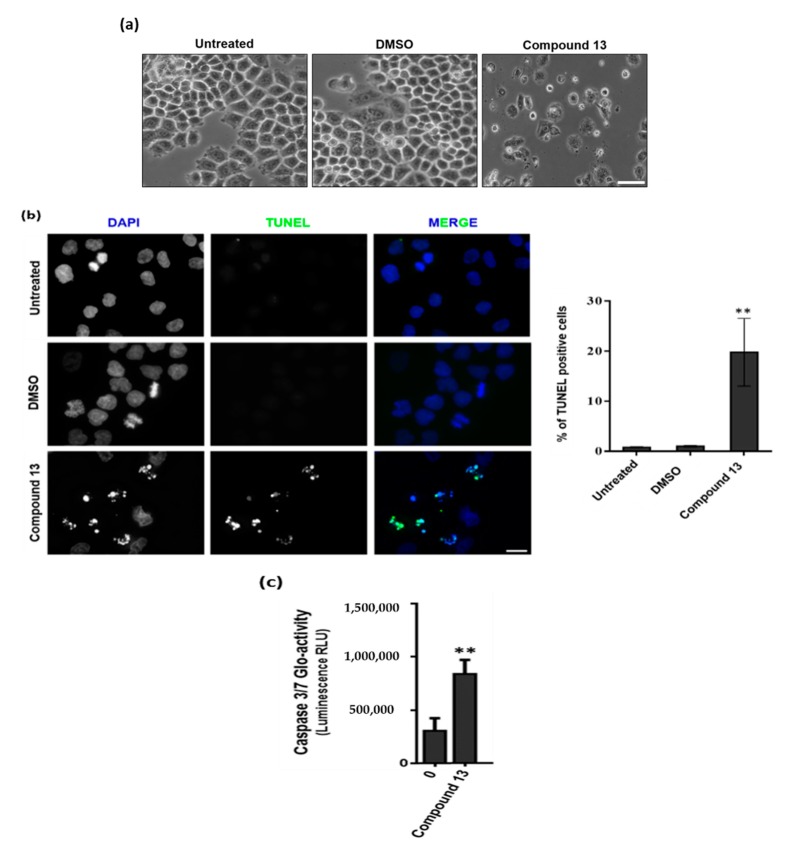
Compound **13** induces apoptotic cell death through caspase 7 activation in MCF-7 cells. (**a**) Representative phase contrast microscopy fields of untreated, 0.04% DMSO- and 4× GI_50_ compound **13**-treated cells for 30 h. Scale bar = 20 µm. (**b**) (**Left**) Cells treated with compound **13** for 30 h and stained with TUNEL to detect apoptotic cells (green). DNA (blue) was stained with DAPI. Untreated- and 0.04% DMSO-treated cells were used as control. Scale bar = 5 µm. (**Right**) Quantification of data shown in left panel. ** *p* = 0.0082 (Unpaired T-test). (**c**) Caspase 3/7 Glo-activity of cells treated with 23.46 µM of compound **13** for 24 h. DMSO-treated cells (0) were used as control. **p = 0.0051 (Unpaired T-test).

The compounds with GI_50_ lower than 10 μM (Table 1) were tested for the ability to induce apoptosis in MCF-7 cells. All compounds were initially tested at concentrations ranging 1–4 times GI_50_ to define their involvement in apoptosis induction. The appearance of apoptosis was monitored up to 30 h from treatment. Treated cells were observed under phase contrast microscope for any morphological changes that occur during apoptosis such as cell detachment, membrane blebbing, chromatin condensation, nuclear fragmentation and formation of apoptotic bodies. After this screening, compound **13** was identified as capable of inducing apoptotic cell morphology in MCF-7 cells, while cells treated with the other compounds were morphologically indistinguishable from the control cultures (Figure 2a). The induction of apoptosis was further confirmed by the TUNEL assay, showing increased number of TUNEL-positive cells with DNA fragmentation (Figure 2b).

Furthermore, the compound was assessed for the ability to induce caspase-3/7 activation, as measured with the proluminescent caspase-3/7 substrate containing the tetrapeptide sequence DEVD. The results show that compound **13** can directly process procaspases-3/7 to the active caspases-3/7 (Figure 2c).

**Figure 3:**
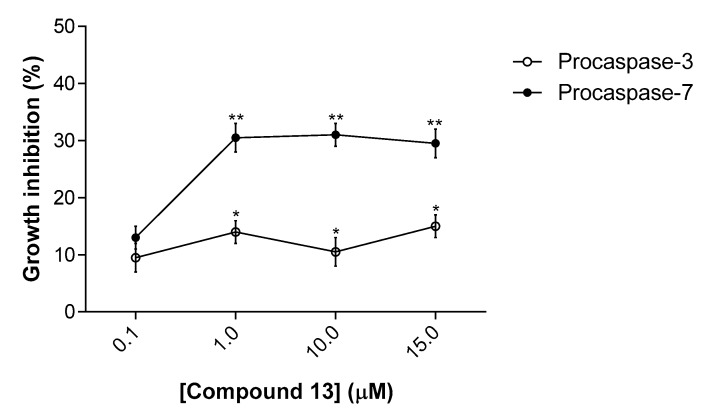
Effect of compound **13** on the growth of yeast cells expressing procaspases-3/7. Concentration-response curves for the effects of compound **13** on the growth of yeast cells expressing human procaspase-7 or procaspase-3, for 24 h treatment. The percentage of drug-induced growth inhibition was estimated considering 100% growth the number of CFU obtained with DMSO only. Data are mean ± SEM of six independent experiments; values significantly different from DMSO are indicated (* *p* < 0.05, ** *p* = 0.001; unpaired t-test).

To further confirm the activity of compound **13** on caspases 3 and 7, we used a yeast cell system previously developed by our group [12]. This assay is based on the ectopic expression of human procaspases-3/7 in yeast, which after processing, namely by a small-molecule activator, lead to an active form of caspase that is cytotoxic in yeast. In this assay, a correlation between the yeast growth inhibition and the degree of activation of human caspase was established [12]. The results obtained show that compound **13** inhibited the growth of yeast cells expressing procaspase-7 at 1–15 µM, exhibiting a much lower growth inhibitory effect on yeast cells expressing procaspase-3 (Figure 3). It is worth noting that compound **13** did not interfere with the growth of control yeast (transformed with the empty vector; data not shown).

**Figure 4:**
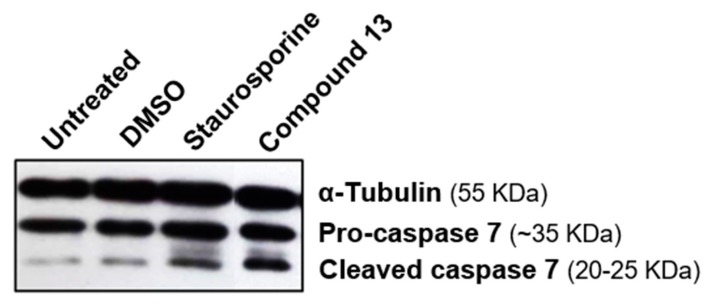
Compound **13** promotes MCF-7 cells growth inhibition associated with caspase 7 activation in MCF-7 cells. Caspase 7 activity as detected by immunoblotting against protein extracts from MCF-7 cells treated with compound **13** for 24 h. DMSO-treated cells and 100 µM Staurosporine (6 h) were included as controls. α-tubulin was used as a loading control.

The results obtained with yeast assays were corroborated by the significant procaspase 7 cleavage activity observed by Western blot analysis against protein extracts from MCF-7 cells treated with compound **13** (Figure 4).

Altogether, the results obtained support that flavonoid **13** is a potential activator of caspase-7 in human tumor cells.

### 2.4. Docking Studies

In this study, compound **13** was discovered as a procaspase-7 activator. Therefore, docking studies were performed for this compound along with compounds already described in the literature as procaspase-7 activators (**29**–**31**) [28,29], which were used as positive controls. For positive controls, docking score values ranging from −6.1 to −6.4 Kcal·mol^−1^ were obtained (Table 2). According to the docking study, **13** forms a more stable complex with procaspase-7, thus presenting a lower docking score (−7.7 Kcal·mol^−1^), than known activators **29**, **30** (−6.1 Kcal·mol^−1^) and **31** (−6.4 Kcal·mol^−1^). These results are in accordance with in vitro studies (Figure 1, Figure 2 and Figure 3).

**Figure 5:**
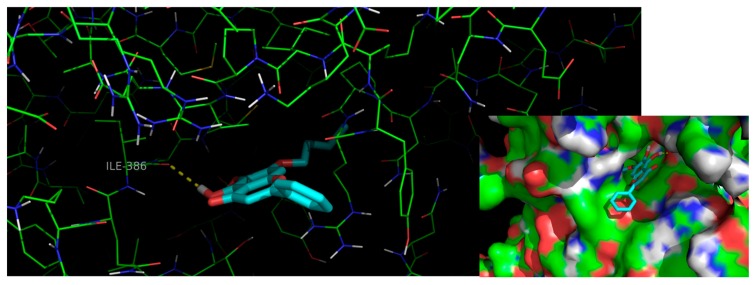
Interactions of flavone **13** (blue sticks) with residues in the allosteric site of procaspase-7. Polar interactions are represented as yellow broken lines. Carbon, oxygen, nitrogen and sulfur atoms of the target are represented in green, red, blue, and yellow, respectively.

To further understand the binding mode of **13** to procaspase-7, a careful inspection of the most stable docking pose of this small molecule was performed (Figure 5).

Compound **13** establishes one hydrogen interaction with procaspase-7 Ile-886 (Figure 5). Moreover, the isopentyloxyl group allows a more favorable orientation and a deeper insertion of the molecule into the allosteric groove of the target, as well as the establishment of additional van der Waals interactions with the hydrophobic cavity.

### 2.5. QSAR Model

Since QSAR studies have been used for decades to highlight small molecules properties and to predict different important biological activities [30], with the overall results obtained for the in vitro growth inhibitory effect in MCF-7 cell lines, a QSAR model was built to highlight the features important for the growth inhibitory activity of these derivatives. This model will enable speeding the design of new active compounds. In this work, a 2D-QSAR model was elaborated using Comprehensive Descriptors for Structural and Statistical Analysis (CODESSA) software package (CODESSA software version 2.7.2, University of Florida, Gainesville, FL, USA). Many constitutional, topological, geometrical, electrostatic and quantum-chemical descriptors were generated. The heuristic method proceeds with a preselection of descriptors by eliminating: those descriptors that are not available for each structure; descriptors having a small variation in magnitude for all structures; descriptors found to be correlated pairwise; and descriptors found to be of no statistical significance. The heuristic method is a very useful tool for searching the best pool of descriptors. It is a quick method and presents no restrictions on the size of the dataset [31].

**Figure 6:**
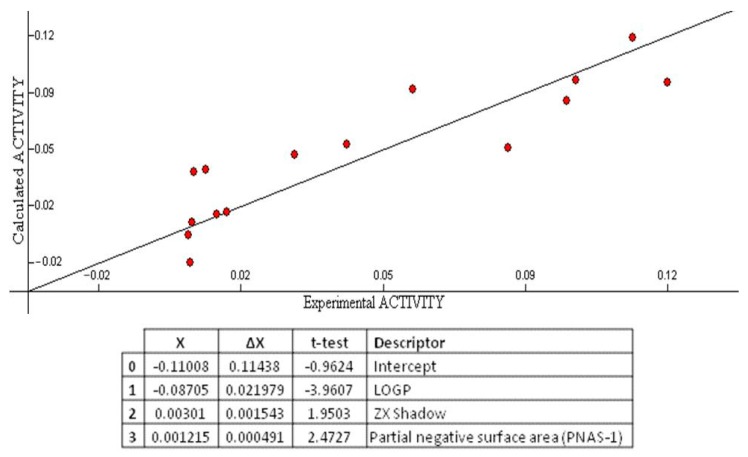
QSAR model obtained with the heuristic method for 15 chalcones with the CODESSA software (*R*^2^ = 0.7446, F = 10.69, and s = 0.0006). X, ΔX and t-test are the regression coefficient of the linear model, standard errors of the regression coefficient, and the *t* significance coefficient of the determination, respectively.

The correlation coefficient (*R*^2^) (a statistical measure of how close the data are to the fitted regression line), standard error(s) (which consists of an absolute measure of the quality of fit), Fisher’s value (F) (which represents the F-ratio between the variance of actual and predicted activity), and cross-validation (*Q*^2^) (which measures the goodness-of-prediction) were employed to judge the validity of regression equation. A major point in developing a QSAR model is the number of descriptors used to elaborate the equation. Laws of QSAR establish that it should be one descriptor for each five molecules [32]. Accordingly, as the training set was composed of 15 molecules, three descriptors were used to build the QSAR model. The multilinear regression analysis using Heuristic method for 15 compounds in the three-parameter model is given in Figure 6.

The best training model had a quality (*R*^2^) of 0.7446, Fisher value of 10.69, and S of 0.0006, which demonstrate that the proposed model has statistical stability and validity despite the small group of molecules used to build the model. The squared correlation coefficient *R*^2^ is a relative measure of quality of fit by regression equation [33]. Correspondingly, it represents more than 70% of the total variance (*R*^2^ = 0.7446) in growth inhibitory activity exhibited by the tested compounds. R^2^ is greater than 0.6, which is an indicator of a good fit to the regression line [34]. The F-test reflects the ratio of the variance explained by the model and the variance due to the error in the regression. High value of the F-test indicates that the model is statistically significant. The QSAR model is significant at 95% level, as shown by their Fischer ratio values, which exceed the tabulated values (3.59) as desired for a meaningful correlation [35]. Standard errors express the variation of the residuals or the variation about the regression line. Thus, standard deviation is an absolute measure of quality of fit and should have a low value for the regression to be significant [36]. The cross-validated *R*^2^ (*Q*^2^) process repeats the regression many times on subsets of data and R is computed using the predicted values of the missing molecules. *Q*^2^ (0.60) is smaller than the overall *R*^2^ (0.74), as expected, but still the difference between *R*^2^ and *Q*^2^ is lower than 0.3, which indicates that the model has good predictive power [34]. External (test set) predictivity was used as validation criterion, and the model was able to predict the growth inhibitory activity with an average difference of 0.03 from the experimental value [37]. From all the above, it can be concluded that the QSAR model is applicable for growth inhibitory activity, which suggests that the model may have predictive capacity for more inhibitors of MCF-7 cell line.

Octanol-water partition coefficient logP, ZX shadows, and partial negative surface area descriptors (PNSA-1) were predicted as being involved in the growth inhibitory activity of the tested compounds (Figure 6).

LogP [38] is included in several QSAR studies and rational drug design as a measure of molecular hydrophobicity. The negative sign suggests that the growth inhibitory activity is inversely related to this descriptor. Therefore, if lipophily increases, it leads to a decrease in activity. For example, **11**, with a butoxy side chain on C-7, is more active than **12** that has two butoxy chains at C-7 and C-6.

ZX Shadow is a geometrical descriptor that reflects the overall shape of the molecule projected onto the plane ZX oriented with respect to its moments of inertia [39]. ZX Shadow depends on its substituent and its position in the flavone ring system. For example, **9** contains an allyl substituent at C-7, thus presenting a different molecular shape than **10**, which has the same substituent in C-6. Therefore, **9** is much more active than **10**.

PNSA-1 (partial negative surface area) is the sum of surface area on negative parts of molecule. PNAS-1 is influenced by the presence of polar atoms such as oxygen and alkene side chains, being therefore related to the ability of molecules to form hydrogen bond. Thus, two hydroxyl groups at C5 and C6 (**7**, **11** and **13**) are more favorable for activity than their absence (**17** and **19**–**21**) or the presence of just one hydroxyl at C5 (**24** and **26**). Alkenes are also slightly more polar than alkanes because the bond electrons are more polarizable, therefore contributing to instantaneous dipole moments, and the vinylic bond tends to be slightly polar, contributing to the permanent dipole moment [40]. Thus, the baicalein derivative **9** with an alkene side chain is slightly more active than the baicalein derivative **7** with an alkane side chain.

In summary, the structure-property relationship captured by the linear model indicates that hydrophilicity, pattern of ring substitution/shape, and presence of partial negative charged atoms dominate the relationship. More polar molecules, with more hydroxyl substituents in the flavone scaffold, and with shorter alkyl side chains at only one position (C-7), are generally more active. It can be foreseen that the size, the shape, the orientation of the molecule in the caspase binding pocket allowing the establishment of polar contacts may be crucial for a good activity (as revealed by the docking study, Figure 5).

## 3. Material and Methods

### 3.1. Synthesis

MW reactions were performed using a glassware setup for atmospheric-pressure reactions and a 100 mL Teflon reactor (internal reaction temperature measurements with a fiber-optic probe sensor) and were carried out using an Ethos MicroSYNTH 1600 MW Labstation from Milestone (Thermo Unicam, Portugal). All reactions were monitored by thin-layer chromatography (TLC). Purifications of compounds were carried out by flash chromatography using Macherey-Nagel silica gel 60 (0.04–0.063 mm), preparative TLC using Macherey-Nagel silica gel 60 (GF254) plates. Melting points were obtained in a Köfler microscope (Wagner and Munz, Munich, Germany) and are uncorrected. ^1^H and ^13^C NMR spectra were taken in CDCl_3_ at room temperature, on Bruker Avance 300 instrument (300.13 MHz for ^1^H and 75.47 MHz for ^13^C, Bruker Biosciences Corporation, Billerica, MA, USA). Chemical shifts are expressed in δ (ppm) values relative to tetramethylsilane (TMS) used as an internal reference; ^13^C NMR assignments were made by 2D (HSQC and HMBC) NMR experiments (long-range ^13^C-1H coupling constants were optimized to 7 Hz). HRMS mass spectra were recorded as ESI (electrospray ionization) mode on MicrOTOF spectrometer (Bruker Corporation, Billerica, MA, USA) at C.A.C.T.I.-University of Vigo, Spain. The commercially available reagents were purchased from Sigma Aldrich Co. (St. Louis, MO, USA). Reagents and solvents were purified and dried according to the usual procedures described elsewhere [41]. The following materials were synthesized and purified by the described procedures.

#### 3.1.1. General Procedure for the Synthesis of Baicalein Derivatives (**4**–**14**)

A mixture of baicalein (**1**) (0.20 g, 0.74 mmol), methyl/ethyl/propyl/allyl/butyl/isopentyl iodide (1.18 mmol) and anhydrous K_2_CO_3_ (0.55 g, 3.7 mmol) in anhydrous acetone (60 mL) was submitted to successive 30 min of microwave irradiation at 200 W of potency. The final temperature was 60 °C and the total irradiation time was 2 h, except for the reaction with isopentyl iodide, for which the reaction time was 3 h. After cooling, the solid was filtered and the solvent removed under reduced pressure to afford the crude product. The yellow-orange solid obtained was dissolved in acetone and purified as described below.

*7-ethoxy-5,6-dihydroxy-2-phenyl-4H-chromen-4-one* (**6**). Purified by flash chromatography (SiO_2_; n-hexane: EtOAc, 9.5:0.5). Yield: 4.5%; mp 159–161 °C; IR (kBr) *v*max: 3600–3300, 2952, 2922, 1653, 1559, 1507, 1457, 1189 cm^−1^; ^1^H NMR (CDCl_3_, 300.13 MHz) *δ* 12.55 (OH, s, H-5), 7.91–7.88 (2H, m, H-2′,-6′), 7.55–7.53 (3H, m, H-3′,-4′,-5′), 6.69 (1H, s, H-3), 6.61 (1H, s, H-8), 4.24 (2H, q, *J* = 14.0, 7.0, H-1′’), 1.55 (3H, t, *J* = 7.0, H-2′’); ^13^C NMR (CDCl_3_, 75.47 MHz) *δ* 182.7 (C4), 164.1 (C2), 152.2 (C7), 150.7 (C8a), 145.7 (C5), 131.8 (C4′), 131.5 (C1′), 129.7 (C6), 129.1 (C3′, 5′), 126.3 (C2′, 6′), 106.0 (C4a), 105.5 (C3), 91.1 (C8), 65.2 (C1′′), 14.6 (C2′′); ESI-TOF-HRMS (+) *m*/*z*: Anal. Calc. for C_17_H_15_O_5_ (M + H^+^): 299.09140; found: 299.09222.

*5,6-dihydroxy-2-phenyl-7-propoxy-4H-chromen-4-one* (**7**). Purified by flash chromatography (SiO_2_; petroleum ether: EtOAc, 8:2). Yield: 3.5%; mp 167–170 °C; IR (kBr) *v*max: 3600–3300, 2927, 2922, 2851, 1617, 1559, 1489, 1473, 1459, 1458 cm^−1^; ^1^H NMR (CDCl_3_, 300.13 MHz) *δ* 12.50 (OH, s, H-5), 7.91–7.88 (2H, m, H-2′, -6′), 7.55–7.52 (3H, m, H-3′, -4′, -5′), 6.69 (1H, s, H-3), 6.61 (1H, s, H-8), 5.37 (OH, s, H-6), 4.12 (2H, t, *J* = 6.9, H-1′′), 2.05–1.98 (2H, m, H-2′′), 1.10 (3H, t, *J* = 7.4, H-3′′); ^13^C NMR (CDCl_3_, 75.47 MHz) *δ* 182.7 (C4), 164.0 (C2), 152.3 (C7), 150.7 (C8a), 145.6 (C5), 131.7 (C4′), 131.6 (C1′), 129.7 (C6), 129.1 (C3′, 5′), 126.2 (C2′, 6′), 106.0 (C4a), 105.4 (C3), 91.1 (C8), 71.0 (C1′′), 22.3 (C2′′), 10.4 (C3′′); ESI-TOF-HRMS (+) *m*/*z*: Anal. Calc. for C_18_H_17_O_5_ (M + H^+^): 313.10705; found: 313.10708.

*5,6-dihydroxy-7-(isopentyloxy)-2-phenyl-4H-chromen-4-one* (**13**). Purified by flash chromatography (SiO_2_; n-hexane: EtOAc, 7:3). Yield: 5.6%; mp 175–178 °C; IR (kBr) *v*max: 3600–3300, 2955, 2921, 2854, 1657, 1489, 1477, 1450, 1115 cm^−1^; ^1^H NMR (CDCl_3_, 300.13 MHz) *δ* 12.79 (OH, s, H-5), 7.91–7.88 (2H, m, H-2′, -6′), 7.54–7.52 (3H, m, H-3′, -4′, -5′), 6.69 (1H, s, H-3), 6.61 (1H, s, H-8), 5.41 (OH, s, H-6), 4.18 (2H, t, *J* = 6.6, H-1′’), 1.93–1.77 (2H, m, H-2′’), 1.29–1.23 (1H, m, H-3′′), 1.01 (6H, d, *J* = 6.3, H-4′′, -5′′); ^13^C NMR (CDCl_3_, 75.47 MHz) *δ* 182.7 (C4), 164.0 (C2), 152.3 (C7), 150.7 (C8a), 145.7 (C5), 131.8 (C4′), 131.5 (C1′), 129.7 (C6), 129.1 (C3′, 5′), 126.3 (C2′, 6′), 106.0 (C4a), 105.4 (C3), 91.1 (C8), 68.1 (C1′′), 37.6 (C2′′), 31.0 (C3′′), 25.1 (C4′′), 22.6 (C5′′); ESI-TOF-HRMS (+) *m*/*z*: Anal. Calc. for C_20_H_21_O_5_ (M + H^+^): 341.13835; found: 341.13857.

*5-hydroxy-6,7-bis(isopentyloxy)-2-phenyl-4H-chromen-4-one* (**14**). Purified by flash chromatography (SiO_2_; n-hexane: EtOAc, 9:1) followed by preparative TLC (SiO_2_; n-hexane: EtOAc, 8:2). Yield: 0.6%; mp 85–87 °C; IR (kBr) *v*max: 3600-3300, 2957, 2922, 2851, 1653, 1559, 1497, 1457, 1419 cm^−1^; ^1^H NMR (CDCl_3_, 300.13 MHz) *δ* 12.61 (OH, s, H-5), 7.91–7.88 (2H, m, H-2′, -6′), 7.54–7.52 (3H, m, H-3′, -4′, -5′), 6.67 (1H, s, H-3), 6.56 (1H, s, H-8), 4.12 (2H, t, *J* = 6.5, H-1′′), 4.06 (2H, t, *J* = 6.9, H-1′′), 1.96–1.84 (2H, m, H-3′′, 3′′′), 1.79 (2H, q, *J* = 6.6, H-2′′), 1.69 (2H, q, *J* = 6.8, H-2′′′), 1.00 (6H, d, *J* = 6.5, H-4′′, -5′′) 0.97 (6H, d, *J* = 6.6, H-4′′′, -5′′′); ^13^C NMR (CDCl_3_, 75.47 MHz) *δ* 182.7 (C4), 163.9 (C2), 158.9 (C7), 153.3 (C8a), 145.7 (C5), 131.9 (C6), 131.7 (C1′), 129.1 (C3′, C5′), 126.2 (C2′, 6′), 106.3 (C4a), 105.6 (C3), 91.2 (C8), 71.8 (C1′′′), 67.5 (C1′′), 38.9 (C2′′′), 37.6 (C2′′), 29.8 (C3′′′), 29.3 (C3′′), 22.7 (C4′′′), 22.6 (C4′′, 5′′′), 22.5 (C5′′); ESI-TOF-HRMS (+) *m*/*z*: Anal. Calc. for C_25_H_31_O_5_ (M + H^+^): 411.21660; found: 411.21677.

#### 3.1.2. General Procedure for the Synthesis of 3,7-Dihydroxyflavone Derivatives (**15**–**21**)

A mixture of 3,7-dihydroxyflavone (**2**) (0.19 g, 0.74 mmol), methyl/ethyl/propyl/allyl/butyl/isopentyl iodide (1.18 mmol) and anhydrous K_2_CO_3_ (0.55 g, 3.7 mmol) in anhydrous acetone (60 mL) was submitted to successive 30 min of MW irradiation at 200 W of potency. Total irradiation time was 2 h and the final temperature was 60 °C. After cooling, the solid was filtered and the solvent removed under reduced pressure to afford the crude product. The yellow-green solid obtained was dissolved in acetone and purified as described below.

*3-hydroxy-2-phenyl-7-propoxy-4H-chromen-4-one* (**17**). Purified by flash chromatography (SiO_2_; petroleum ether: EtOAc, 95:5) followed by preparative TLC (SiO_2_; n-hexane: EtOAc, 8:2) and crystallization (chloroform: n-hexane). Yield: 3.7% as yellow crystals; mp 173–174 °C; IR (kBr) *v*max: 3600–3300, 2970, 1920, 1603, 1576, 1504, 1432, 1412, 1259 cm^−1^; ^1^H NMR (CDCl_3_, 300.13 MHz) *δ* 8.25–8.22 (2H, m, H-2′, -6′), 8.13 (1H, d, *J* = 8.9, H-5), 7.61–7.43 (3H, m, H-3′, -4′, -5′). 7.00 (1H, dd, *J* = 10.4, 2.3, H-6), 6.97 (1H, d, *J* = 2.3, H-8), 4.05 (2H, t, *J* = 6.6, H-1′′), 1.95–1.83 (2H, m, H-2′′), 1.09 (3H, t, *J* = 7.4, H-3′′); ^13^C NMR (CDCl_3_,75.47 MHz) *δ* 172.9 (C4), 163.9 (C7), 157.4 (C8a), 144.1 (C2), 138.1 (C3), 131.3 (C1′),129.9 (C4′), 128.6 (C3′, 5′), 127.5 (C2′, 6′), 126.7 (C5), 115.3 (C6), 114.4 (C4a), 100.3 (C8), 70.3(C1′′), 22.4 (C2′′), 10.5 (C3′′); ESI-TOF-HRMS (+) *m*/*z*: Anal. Calc. for C_18_H_17_O_4_ (M + H^+^): 297.11214; found: 297.11218.

*7-(allyloxy)-3-hydroxy-2-phenyl-4H-chromen-4-one* (**18**). Purified by flash chromatography (SiO_2_; n-hexane: EtOAc, 9.75:0.25) followed by preparative TLC (SiO_2_; n-hexane: EtOAc, 8:2) and crystallization (chloroform: n-hexane). Yield: 9.3%; mp 144–146 °C; IR (kBr) *v*max: 3600–3300, 2999, 2964, 2921, 2847, 1615, 1564, 1503, 1473, 1453, 1260 cm^−1^; ^1^H NMR (CDCl_3_, 300.13 MHz) *δ* 8.22–8.16 (2H, m, H-2′, -6′), 8.13 (1H, d, *J* = 8.9, H-5), 7.03 (1H, dd, *J* = 8.6, 2.3, H-6), 6.97 (1H, d, *J* = 2.3, H-8), 6.16–6.03 (1H, m, H-2′′), 5.52–5.44 (2H, m, H-3′′a), 5.40–5.31 (2H, m, H-3′′b), 4.67 (2H, dt, *J* = 5.3, 1.5, H-1′′); ^13^C NMR (CDCl_3_, 75.47 MHz) *δ* 172.8 (C4), 163.2 (C7), 157.3 (C8a), 144.1 (C2), 138.1 (C3),131.9 (C2′’) 131.2 (C1′), 129.9 (C4′), 128.6 (C3′, 5′), 127.5 (C2′, 6′), 126.8 (C5),118.6 (C3′′), 115.2 (C6), 100.8 (C8), 69.4 (C1′′); ESI-TOF-HRMS (+) *m*/*z*: Anal. Calc. for C_18_H_15_O_4_ (M + H^+^): 295.09649; found: 295.09689.

*7-butoxy-3-hydroxy-2-phenyl-4H-chromen-4-one* (**19**). Purified by flash chromatography (SiO_2_; n-hexane: EtOAc, 9.5;0.5) followed by preparative TLC (SiO_2_; n-hexane: EtOAc, 8:2) and crystallization (chloroform: n-hexane) Yield: 9.7%; mp 149–151 °C; IR (kBr) *v*max: 3600–3300, 2958, 2922, 2854, 1604, 1566, 1462, 1452, 1419, 1250 cm^−1^; ^1^H NMR (CDCl_3_, 300.13 MHz) *δ* 8.25–8.22 (2H, m, H-2′, -6′), 8.13 (1H, d, *J* = 8.6, H-5), 7.56–7.43 (3H, m, H-3′, -4′, -5′), 7.00 (1H, dd, *J* = 8.8, 2.3, H-6), 6.95 (1H, d, *J* = 2.3, H-8), 4.09 (2H, t, *J* = 6.5, H-1′′), 1.89–1.80 (2H, m, H-2′′), 1.60–1.47 (2H, m, H-3′′), 1.01 (3H, t, *J* = 7.4, H-4′′); ^13^C NMR (CDCl_3_, 75.47 MHz) *δ* 206.0 (C4), 162.9 (C7), 156.4 (C8a), 143.1 (C2), 137.1 (C3), 131.3 (C1′), 130.2 (C4′), 128.9 (C3′, 5′), 127.5 (C2′, 6′), 126.5 (C5), 114.2 (C6), 113.4 (C4a), 99.3 (C8), 67.4 (C1′′), 29.9 (C2′′), 18.2 (C3′′), 12.8 (C4′′); ESI-TOF-HRMS (+) *m*/*z*: Anal. Calc. for C_19_H_19_O_4_ (M + H^+^): 311.12779; found: 311.12736.

*3,7-dibutoxy-2-phenyl-4H-chromen-4-one* (**20**). Purified by flash chromatography (SiO_2_; n-hexane: EtOAc, 9.75:0.25). Yield: 7.1%; mp 190–193 °C; IR (kBr) *v*max: 3600–3300, 2936, 1873, 1623, 1499, 1466, 1447, 1260 cm^−1^; ^1^H NMR (CDCl_3_, 300.13 MHz) *δ* 8.10–8.07 (2H, m, H-2′, -6′), 8.14 (1H, d, *J* = 8.9, H-5), 7.52–7.49 (3H, m, H-3′, -4′, -5′), 6.96 (1H, dd, *J* = 8.9, 2.3, H-6), 6.90 (1H, d, *J* = 2.3, H-8), 4.06 (2H, t, *J* = 6.5, H-1′′), 4.02 (2H, t, *J* = 6.5, H-1′′′), 1.87–1.78 (2H, m, H-2′′), 1.73–1.63 (2H, m, H-2′′′), 1.59–1.49 (2H, m, H-3′′), 1.42–1.32 (2H, m, H-3′′), 1.00 (3H, t, *J* = 7.4, H-4′′), 0.87 (3H, t, *J* = 7.4, H-4′′′); ^13^C NMR (CDCl_3_, 75.47 MHz) *δ* 174.8 (C4), 163.6 (C7), 157.1 (C8a), 155.3 (C2), 140.5 (C3), 131.3 (C1′), 130.6 (C4′), 128.3 (C2′, 6′), 128.7 (C3′. C5′), 127.1 (C5), 117.9 (C6), 114.8 (C4a), 100.3 (C8), 72.6 (C1′’’), 68.4 (C1′′), 32.1 (C2′′′), 31.0 (C2′′), 19.2 (C3′′), 19.1 (C3′′), 13.8 (C4′′, 4′′′); ESI-TOF-HRMS (+) *m*/*z*: Anal. Calc. for C_23_H_27_O_4_ (M + H^+^): 367.19039; found: 367.19036.

*3-hydroxy-7-(isopentyloxy)-2-phenyl-4H-chromen-4-one* (**21**). Purified by flash chromatography (SiO_2_; petroleum ether: EtOAc, 9.5:0.5) followed by preparative TLC (SiO_2_; n-hexane: EtOAc, 8:2) and crystallization (chloroform: n-hexane). Yield: 12.9%; mp 181–184 °C; IR (kBr) *v*max: 3600–3300, 2958, 2921, 2854, 1605, 1504, 1467, 1452, 1410, 1260 cm^−1^; ^1^H NMR (CDCl_3_, 300.13 MHz) *δ* 8.25–8.22 (2H, m, H-2′, -6′), 8.12 (1H, d, *J* = 8.9, H-5), 7.56–7.42 (3H, m, H-3′, -4′, -5′), 6.98 (1H, dd, *J* = 8.9, 2.3, H-6), 6.94 (1H, d, *J* = 2.3, H-8), 4.11 (2H, t, *J* = 6.6, H-1′’), 1.92–1.81 (1H, m, H-3′’), 1.78-1.72 (2H, m, H-2′’), 1.00 (6H, d, *J* = 6.5, H-4′′, -5′′); ^13^C NMR (CDCl_3_, 75.47 MHz) *δ* 172.9 (C4), 163.9 (C7), 157.4 (C8a), 144.1 (C2), 138.1 (C3), 131.3 (C1′), 129.9 (C4′), 128.6 (C3′, 5′), 127.5 (C2′, 6′), 126.7 (C5), 115.3 (C6), 114. (C4a), 100.3 (C8), 67.2 (C1′′), 37.6 (C2′′), 25.1 (C3′′), 22.6 (C4′′, 5′′); ESI-TOF-HRMS (+) *m*/*z*: Anal. Calc. for C_20_H_21_O_4_ (M + H^+^): 325.14344; found: 325.14280.

#### 3.1.3. General Procedure for the Synthesis of Chrysin Derivatives (**22**–**28**)

A mixture of chrysin (**3**) (0.188 g, 0.74 mmol), methyl/ethyl/propyl/allyl/butyl/isopentyl iodide (1.18 mmol) or prenyl bromide (171 µL, 1.48 mmol) and anhydrous K_2_CO_3_ (0.55 g, 3.7 mmol) in anhydrous acetone (60 mL) was submitted to successive 30 min of MW irradiation at 200 W of potency. Total irradiation time was 90 min and the final temperature was 60 °C. After cooling, the solid was filtered and the solvent removed under reduced pressure to afford the crude product. The solid obtained was dissolved in acetone and purified by flash chromatography (SiO_2_; n-hexane: EtOAc; 9:1).

*5-hydroxy-2-phenyl-7-propoxy-4H-chromen-4-one* (**24**). Yield: 22%; mp 132–135 °C; IR (kBr) *v*max: 3600–3400, 2964, 2920, 2874, 1661, 1585, 1569, 1507, 1451, 1173 cm^−1^; ^1^H NMR (CDCl_3_, 300.13 MHz) *δ* 12.70 (OH, s, H-5), 7.90–7.87 (2H, m, H-2′, -6′), 7.56–7.49 (3H, m, H-3′, -4′, -5′), 6.66 (1H, s, H-3), 6.50 (1H, d, *J* = 2.2, H-8), 6.37 (1H, d, *J* = 2.2, H-6), 4.00 (2H, t, *J* = 6.6, H-1′′), 1.90–1.79 (2H, m, H-2′′), 1.06 (3H, t, *J* = 7.4, H-3′′); ^13^C NMR (CDCl_3_,75.47 MHz) *δ* 182.5 (C4), 165.2 (C7), 163.9 (C2), 162.1 (C5), 157.8 (C8a), 131.8 (C4′), 131.4 (C1′), 129.1 (C3′, 5′), 126.3 (C2′, 6′), 105.9 (C3), 105.6 (C4a), 98.6 (C6), 93.1 (C8), 70.2 (C1′′), 22.3 (C2′′), 10.4 (C3′′); ESI-TOF-HRMS (+) *m*/*z*: Anal. Calc. for C_18_H_17_O_4_ (M + H^+^): 297.11214; found: 297.11210.

*5-hydroxy-7-(isopentyloxy)-2-phenyl-4H-chromen-4-one* (**27**). Yield: 30%; mp 129–132 °C; IR (kBr) *v*max: 3600–3400, 2956, 2921, 1851, 1662, 1588, 1452, 1169 cm^−1^; ^1^H NMR (CDCl_3_, 300.13 MHz) *δ* 12.71 (OH, s, H-5), 7.90–7.87 (2H, m, H-2′, -6′), 7.55–7.52 (3H, m, H-3′, -4′, -5′), 6.67 (1H, s, H-3), 6.50 (1H, d, *J* = 2.2, H-8), 6.37 (1H, d, *J* = 2.2, H-6), 4.06 (2H, t, *J* = 6.6, H-1′’), 1.92-1.78 (1H, m, H-3′′), 1.75–1.68 (2H, m, H-2′′), 0.98 (6H, d, *J* = 6.5, H-4′′, 5′′); ^13^C NMR (CDCl_3_, 75.47 MHz) *δ* 182.5 (C4), 168.2 (C7), 163.9 (C2), 162.1 (C5), 158.0 (C8a) 131.8 (C1′), 129.1 (C3′, 5′), 126.3 (C2′, 6′), 105.9 (C3), 98.6 (C6), 93.1 (C8), 67.1 (C1′′), 37.6 (C2′′), 25.0 (C3′′), 22.5 (C4′′, 5′′); ESI-TOF-HRMS (+) *m*/*z*: Anal. Calc. for C_20_H_21_O_4_ (M + H^+^): 325.14344; found: 325.14247.

### 3.2. Biological Activity

#### 3.2.1. Chemicals

All tested compounds were dissolved in dimethyl sulfoxide (DMSO, Sigma-Aldrich, St. Louis, MO, USA) to a stock concentration of 60 mM and stored at −20 °C, in different aliquots. Prior to each assay, the compounds were freshly prepared to the desired concentrations.

#### 3.2.2. Tumor Cell Growth Assay

The human tumor cell lines, A375-C5 (melanoma), MCF-7 (breast adenocarcinoma), and NCI-H460 (non-small cell lung cancer) (European Collection of Cell Culture, Salisbury, Wiltshire, UK), were grown in RPMI-1640 (Biochrom, Berlin, Germany) supplemented with 5% heat-inactivated fetal bovine serum (FBS, Biochrom). All cell lines were maintained at 37 °C in a 5% CO_2_ humidified atmosphere (Hera Cell, Heraeus, Hanau, Germany). Cell viability was routinely determined with trypan blue (Sigma-Aldrich) exclusion assay. All experiments were performed with exponentially growing cells, revealing more than 95% viability. The effect of the compounds under study on cell growth was determined according to the procedure adopted by the National Cancer Institute (NCI) in the “In Vitro Anticancer Drug Discovery Screen”, which uses the protein-binding dye SRB to evaluate cell growth. Accordingly, cells were plated in 96-well plates at a density of 0.05 × 10^6^ cells/well in complete culture medium and incubated at 37 °C. Twenty-four hours later, cells were treated with two-fold serial dilutions of the tested compounds, ranging from 0 to 150 µM. Control groups received the same amount of sterile DMSO (Sigma-Aldrich), used as compounds solvent, up to 0.25% concentration. Forty-eight hours later, cells were fixed in situ with 50% (*m*/*v*) trichloroacetic acid (Merck Millipore, Billerica, MA, USA), washed with distilled water and then stained with SRB (Sigma-Aldrich) for 30 min at room temperature. SRB-stained cells were washed 5 times with 1% (*v*/*v*) acetic acid (Merck Millipore) and left to dry at room temperature. Solubilization of SRB complexes was achieved by adding 10 mM Tris buffer (Sigma-Aldrich) for 30 min at room temperature. Absorbance was measured at 515 nm in a microplate reader (Biotek Synergy 2, Winooski, VT, USA). A dose-response curve was obtained for each cell line with each tested compound and the concentration that consented a 50% cell growth inhibition (GI_50_) was determined.

#### 3.2.3. TUNEL Assay

To evaluate apoptosis induction, terminal deoxynucleotidyl transferase-mediated nick and labelling (TUNEL) assay was performed using the DeadEnd Fluorometric TUNEL System kit (Promega, Madison, WI, USA). MCF-7 cells were treated with 4 times GI_50_ of the drug for 30 h, and then subjected to TUNEL assay according to the manufacturer’s instructions. At the end, DNA was stained with 2 µg/mL of DAPI in Vectashield mounting medium (Vector, H-1000, Burlingame, CA, USA). The number of cells undergoing apoptosis was ascertained by scoring the number of TUNEL-positive cells in a total of 500 cells under fluorescence microscopy, from at least ten randomly selected microscopic fields, for each experimental condition.

#### 3.2.4. Caspase-Glo 3/7 Assay

A total of 0.05 × 10^6^ MCF-7 cells were seeded, in duplicate, into 96-well plates. Twenty-four hours later, cells were treated with 4-time GI_50_ of the drug for 30 h. At the time of apoptosis measurements, cells were incubated with 100 µL of Caspase-Glo 3/7 reagents (Promega) and gently mixed using a multi-channel pipette. Luminescence was measured in a microplate reader (Biotek Synergy 2), at room temperature, up to 3 h. Caspase activity was determined using raw values of luminescence to obtain a relative to control value. The final caspase activity was calculated by averaging two replicates from two independent experiments.

#### 3.2.5. Microscopy Analysis and Image Processing

Phase-contrast microscopy images were obtained with a 10× objective, on a Nikon TE 2000-U microscope (Amsterdam, Netherlands), using a DXM1200F digital camera (Amsterdam, Netherlands) and with Nikon ACT.1 software (version 2.62, Melville, NY, USA). Fluorescence images were acquired with Plan Apochromatic 63×/NA1,4 objective on an Axio Observer Z.1 SD microscope (Carl Zeiss, Germany), coupled to an AxioCam MR3. Z-stacks were acquired with 0.4 µm intervals and after image deconvolution with AxioVision Release SPC software (version 4.8.2, Carl Zeiss, Germany) they were processed using ImageJ (version 1.44, Rashand, W.S., ImageJ, U. S. National Institutes of Health, Bethesda, MD, USA).

#### 3.2.6. Yeast Caspase Assay

*Sacharomyces cerevisiae* expressing human procaspase-3 or -7 was obtained in previous work [12]. For expression of human proteins (routinely grown in minimal selective medium), yeast cells were diluted to 0.05 optical density at 600 nm (OD_600_) in induction selective medium with 2% (*w*/*w*) galactose (Sigma-Aldrich, Sintra, Portugal), 1% glycerol (Sigma-Aldrich), 0.7% (*w*/*w*) yeast nitrogen base without amino acids from Difco (Quilaban, Sintra, Portugal), and all the amino acids required for yeast growth (50 µg/mL) except leucine, and incubated at 30 °C, under continuous orbital shaking (200 rpm). Yeast cells were incubated at 30 °C under continuous orbital shaking (200 rpm) with 0.1–15 µM compound **13** or 0.1% DMSO only, for approximately 24 h (time required by the yeast transformed with the empty vector, control yeast, to achieve 0.3 OD_600_). Yeast growth was analyzed by counting the number of colony-forming units (CFU) after two-day incubation at 30 °C on Sabouraud Dextrose Agar from Liofilchem (Frilabo, Porto, Portugal). For each culture, the percentage of drug-induced growth inhibition was estimated considering 100% growth the number of CFU obtained with yeast incubated with DMSO only.

#### 3.2.7. Western Blotting

Total cell protein extracts were harvested by centrifugation and incubated on ice for 20 min in lysis buffer (50 mM Tris pH 7.5; 150 mM NaCl; 1 mM EDTA; 1% Triton-100) containing a protease inhibitor cocktail (Sigma-Aldrich). The lysates were centrifuged for 5 min at 13000 G, the supernatants collected, and the protein content quantified using the BCA Protein Assay Reagent (Pierce Biotechnology, Waltham, MA, USA), according to the manufacturer’s instructions. Fifteen micrograms of protein lysates were resuspended in SDS-sample buffer (375 mM Tris pH 6.8; 12% SDS; 60% Glycerol; 0.12% Bromophenol Blue; 600 nM DTT), boiled for 3 min at 100 °C, loaded and separated in a 12% SDS-PAGE gel. Proteins were transferred by electro blotting to nitrocelulose membranes (Amersham, UK) by semidry transfer system (Hoefer, Inc., Holliston, MA, USA). The membranes containing the proteins were blocked with 5% non-fat dry milk diluted in TBST (50 mM Tris pH 7.5; 150 mM NaCl, 0.05% Tween-20) for 1 h at room temperature with mild agitation. Primary antibodies were diluted in 1% non-fat dried milk and incubated overnight at 4 °C in agitation. The primary antibodies used were: rabbit anti-caspase 7 (dilution 1:1000, Santa Cruz Biotechnology, Dallas, TX, USA) and mouse anti-α-tubulin (dilution 1:5000, T568 Clone B-5-1-2, Sigma Aldrich). The next day, the membranes were washed and probed with horseradish peroxidase (HRP)-conjugated secondary antibodies diluted at 1:1500 (anti-mouse, Vector) or at 1:1000 (anti-rabbit, Sigma-Aldrich), for 1 h at room temperature.

#### 3.2.8. Statistical Analysis

Statistical analysis was performed using a two-way ANOVA with Tukey’s multiple comparisons test, in GraphPad Prism version 6 (GraphPad software Inc., San Diego, CA, USA). Alpha value was 0.05 and the confidence interval 95%. Data are presented as the means ± standard deviation (SD) of at least three independent experiments. Values of differences with *p* < 0.05 were considered significant.

### 3.3. Virtual Screening and Docking Studies

Structure files for each molecule (**13** and controls **29**–**31**) were created and minimized using the chemical structure drawing tool Hyperchem 7.5 (Hypercube, Gainesville, FL, USA). Docking studies were performed using Autodock Vina software package (Molecular Graphics Lab, San Diego, CA, USA). The molecular modeling program UCSF Chimera 1.4 was used to prepare the receptor (procaspase-7, pdb ID 1GQF). The allosteric site in the procaspase dimer interface [42] was selected for use in docking simulation by building a grid box with the dimensions 25 Å × 25 Å × 25 Å. Docking was performed by incorporating ligand flexibility, and docking scores of the top-ranked poses of each molecule were used for analysis. PyMol1.3 (Schrödinger, New York, NY, USA) was used for visual inspection of results and graphical representations.

### 3.4. QSAR Model

Nineteen tested compounds with GI_50_ on MCF-7 cell line were used to construct a QSAR model using the biological data obtained from the in vitro studies (Growth Inhibitory Activity = −log(1 − (1/GI_50_))), which was adopted as a dependent variable in the QSAR analysis. The 19 molecules were randomly distributed into a training set (15 molecules) and a test set (4 molecules). CODESSA software (version 2.7.10, University of Florida, Gainesville, FL, USA) was used to calculate more than 500 constitutional, topological, geometrical, electrostatic, quantum-chemical and thermodynamical molecular descriptors [43]. The heuristic multilinear regression procedures available in the framework of the CODESSA program was used to perform a complete search for the best multilinear correlations with a multitude of descriptors of the training set. The 2D-QSAR model with the best correlation coefficient (*R*^2^), F-test (F), and standard error (s) was selected. The final model was further validated using the external test set and cross-validation (*Q*^2^).

## 4. Conclusions

In this work, twenty-five flavonoids (**4**–**28**) were synthesized, being compounds **6**, **7**, **13**, **14**, **17**–**21**, **24**, and **27** described here for the first time. The study of their effect on the in vitro cell growth inhibition of human tumor cell lines resulted in the identification of eight hit compounds (**6**, **7**, **9**, **11**, **13**, **15**, **17** and **18**). Among these compounds, **13** was identified as a potential caspase-7 activator. The hydrophilicity, pattern of ring substitution/shape, and presence of partial negative charged atoms seems to influence the growth inhibitory effect of these compounds. The results obtained from this study will be valuable for the rational design of novel and potent caspase-7 activators.

## Figures and Tables

**Table 1:** Effect of flavonoids **4**–**28** on the growth of human tumor cell lines.

Compounds	GI_50_ (μM)		
	A375-C5	MCF7	NCI H460
**4**	>150	>150	>150
**5**	72.05 ± 5.95	78.05 ± 4.85	78.60 ± 3.40
**6**	10.78 ± 1.66	7.86 ± 0.87	7.64 ± 0.18
**7**	7.13 ± 0.76	4.9 ± 0.76	5.10 ± 0.71
**8**	>150	>150	>150
**9**	4.92 ± 1.89	4.37 ± 0.6	4.35 ± 0.4
**10**	80.12 ± 3.73	89.16 ± 4.47	100.3 ± 0.85
**11**	3.61 ± 0.54	3.54 ± 0.54	3.39 ± 1.37
**12**	110.64 ± 19.74	87.07 ± 2.81	76.52 ± 12.83
**13**	5.6 ± 1.94	5.01 ± 0.84	5.06 ± 1.03
**14**	122.50 ± 4.50	>150	>150
**15**	5.68 ± 0.74	5.79 ± 0.04	5.89 ± 0.48
**16**	28.68 ± 24.01	31.71 ± 38.52	38.10 ± 45.32
**17**	9.10 ± 3.23	10.61 ± 1.41	8.26 ± 0.61
**18**	3.35 ± 0.25	4.10 ± 2.20	3.17 ± 0.64
**19**	15.08 ± 0.56	14.9 ± 1.32	14.57 ± 0.36
**20**	103.49 ± 2.84	94.94 ± 1.14	99.49 ± 7.52
**21**	80.97 ± 55.34	80.36 ± 56.75	44.92 ± 8.74
**22**	>150	>150	>150
**23**	52.81 ± 3.17	32.23 ± 0.93	38.7 ± 2.02
**24**	124.71 ± 9.2	106.37 ± 21.54	98.08 ± 20.4
**25**	70.15 ± 18.3	51.59 ± 14.59	58.17 ± 3.01
**26**	>150	>150	>150
**27**	>150	>150	>150
**28**	62.2 ± 16.15	39.33 ± 6.04	33.9 ± 3.67

**Table 2:** Docking scores (Kcal·mol^−1^) of compound **13** and known procaspase activators **29**–**31** using procaspase-7 as target.

Ligand	Docking Scores (Kcal·mol^−1^)
**13**	−7.7
29 *	−6.1
30 *	−6.1
31 *	−6.4

* Used as positive controls for procaspase-7, according to the activation capacity previously described in the literature for those targets [28,29].

## Supplementary Materials

The following are available online, Figure S1. ^1^H and ^13^C NMR of compound **6**, Figure S2. ^1^H and ^13^C NMR of compound **7**, Figure S3. ^1^H and ^13^C NMR of compound **13**, Figure S4. ^1^H and ^13^C NMR of compound **14**, Figure S5. ^1^H and ^13^C NMR of compound **17**, Figure S6. ^1^H and ^13^C NMR of compound **18**, Figure S7. ^1^H and ^13^C NMR of compound **19**, Figure S8. ^1^H and ^13^C NMR of compound **20**, Figure S9. ^1^H and ^13^C NMR of compound **21**, Figure S10. ^1^H and ^13^C NMR of compound **24**, Figure S11. ^1^H and ^13^C NMR of compound **27**, Figure S12. HRMS for compound **6**, Figure S13. HRMS for compound **7**, Figure S14. HRMS for compound **13**, Figure S15. HRMS for compound **14**, Figure S16. HRMS for compound **17**, Figure S17. HRMS for compound **18**, Figure S18. HRMS for compound **19**, Figure S19. HRMS for compound **20**, Figure S20. HRMS for compound **21**, Figure S21. HRMS for compound **24**, Figure S22. HRMS for compound **27**.
