# Supplementary material for: New Alkoxy Flavone Derivatives Targeting Caspases: Synthesis and Antitumor Activity Evaluation
Source: Molecules. 2018 Dec 31;24(1):129. doi: 10.3390/molecules24010129 (PMC6337158; doi:10.3390/molecules24010129)
Supplement: Supplementary file 1 [file molecules-24-00129-s001.pdf]

SUPPORTING INFORMATION

New Alkoxy Flavone Derivatives Targeting Caspases: Synthesis and Antitumor Activity Evaluation

Joana Moreira[1,2], Diana Ribeiro[3], Patrícia M. A. Silva[3], Nair Nazareth[4], Madalena Monteiro[4], Andreia Palmeira[1,2], Lucília Saraiva[4], Madalena Pinto[1,2], Hassan Bousbaa[2,3,*], Honorina Cidade[1,2,*]

[1]Laboratory of Organic and Pharmaceutical Chemistry, Department of Chemical Sciences, Faculty of Pharmacy, University of Porto, Rua Jorge Viterbo Ferreira, 228, 4050-313 Porto, Portugal; joanamoreira.mqf@gmail.com (J. M.); andreiapalmeira@gmail.com (A. P.); madalena@ff.up.pt (M. P.)
[2]Interdisciplinary Centre of Marine and Environmental Research (CIIMAR), University of Porto, Terminal de Cruzeiros do Porto de Leixões, Av. General Norton de Matos s/n 4450-208 Matosinhos, Portugal
[3]CESPU, Institute of Research and Advanced Training in Health Sciences and Technologies (IINFACTS), Rua Central de Gandra, 1317, 4585-116 Gandra, Portugal; diana.ribeiro@cespu.pt (D. R.); patricia.silva@cespu.pt (P. S.)

[4]LAQV/REQUIMTE, Laboratory of Microbiology, Department of Biological Sciences, Faculty of Pharmacy, University of Porto, Rua Jorge Viterbo Ferreira, 228, 4050-313 Porto, Portugal; naircampos@gmail.com (N.N.); madsmonteiro@gmail.com (M.M.); lucilia.saraiva@ff.up.pt (L. S.)
*Correspondence: hcidade@ff.up.pt (H. C.); hassan.bousbaa@iucs.cespu.pt (H. B.); Tel.: +351-220428688; +351-224157186

Table of Contents

NMR spectra……………………………………………………………………...…....3

Figure S1. ^{1}H and ^{13}C NMR of compound **6** ……………………………….………..3

Figure S2. ^{1}H and ^{13}C NMR of compound **7** ………………………………….……....4

Figure S3. ^{1}H and ^{13}C NMR of compound **13** ……………………………..………..5

Figure S4. ^{1}H and ^{13}C NMR of compound **14** …………………………….………....6

Figure S5. ^{1}H and ^{13}C NMR of compound **17** ……………………………….…...….7

Figure S6. ^{1}H and ^{13}C NMR of compound **18** …………………………….…….…..8

Figure S7. ^{1}H and ^{13}C NMR of compound **19**……………………….................9

Figure S8. ^{1}H and ^{13}C NMR of compound **20** ……………………….……......…10

Figure S9. ^{1}H and ^{13}C NMR of compound **21** ......…………………….....................11

Figure S10. ^{1}H and ^{13}C NMR of compound **24** …………...…………….……......12

Figure S11. ^{1}H and ^{13}C NMR of compound **27** ……………………….........13

HRMS spectra ……………………………...……………………...……....14

Figure S12. HRMS of compound **6** 14
Figure S13. HRMS of compound **7** 15
Figure S14. HRMS of compound **13** 16
Figure S15. HRMS of compound **14** 17
Figure S16. HRMS of compound **17**...18
Figure S17. HRMS of compound **18** 19
Figure S18. HRMS of compound **19** 20
Figure S19. HRMS of compound **20** 21
Figure S20. HRMS of compound **21** 22
Figure S21. HRMS of compound **24** 23
Figure S22. HRMS of compound **27** 24

NMR spectra

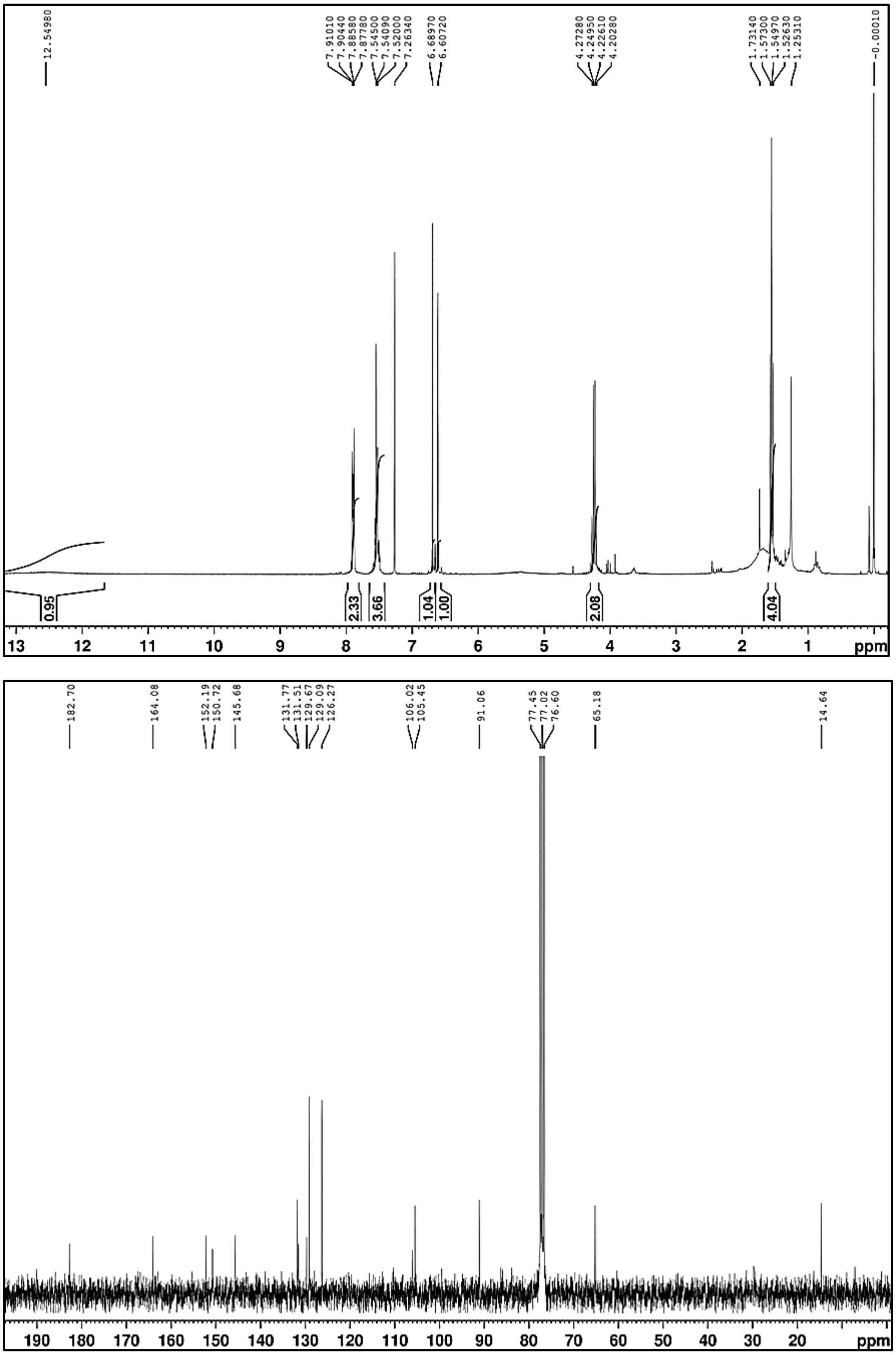

Figure S1. ^{1}H and ^{13}C NMR of compound **6**.

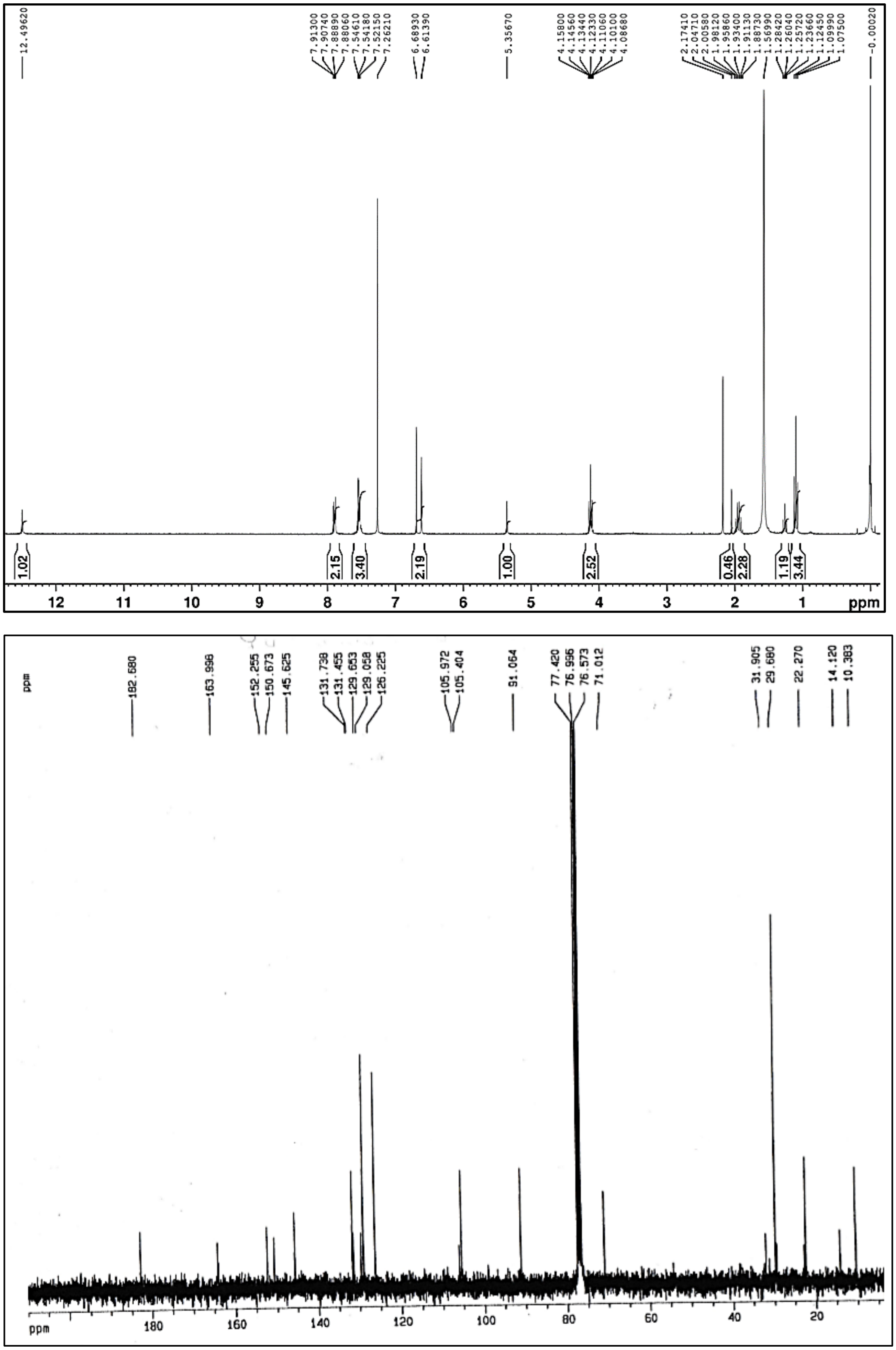

Figure S2. ^{1}H and ^{13}C NMR of compound **7**.

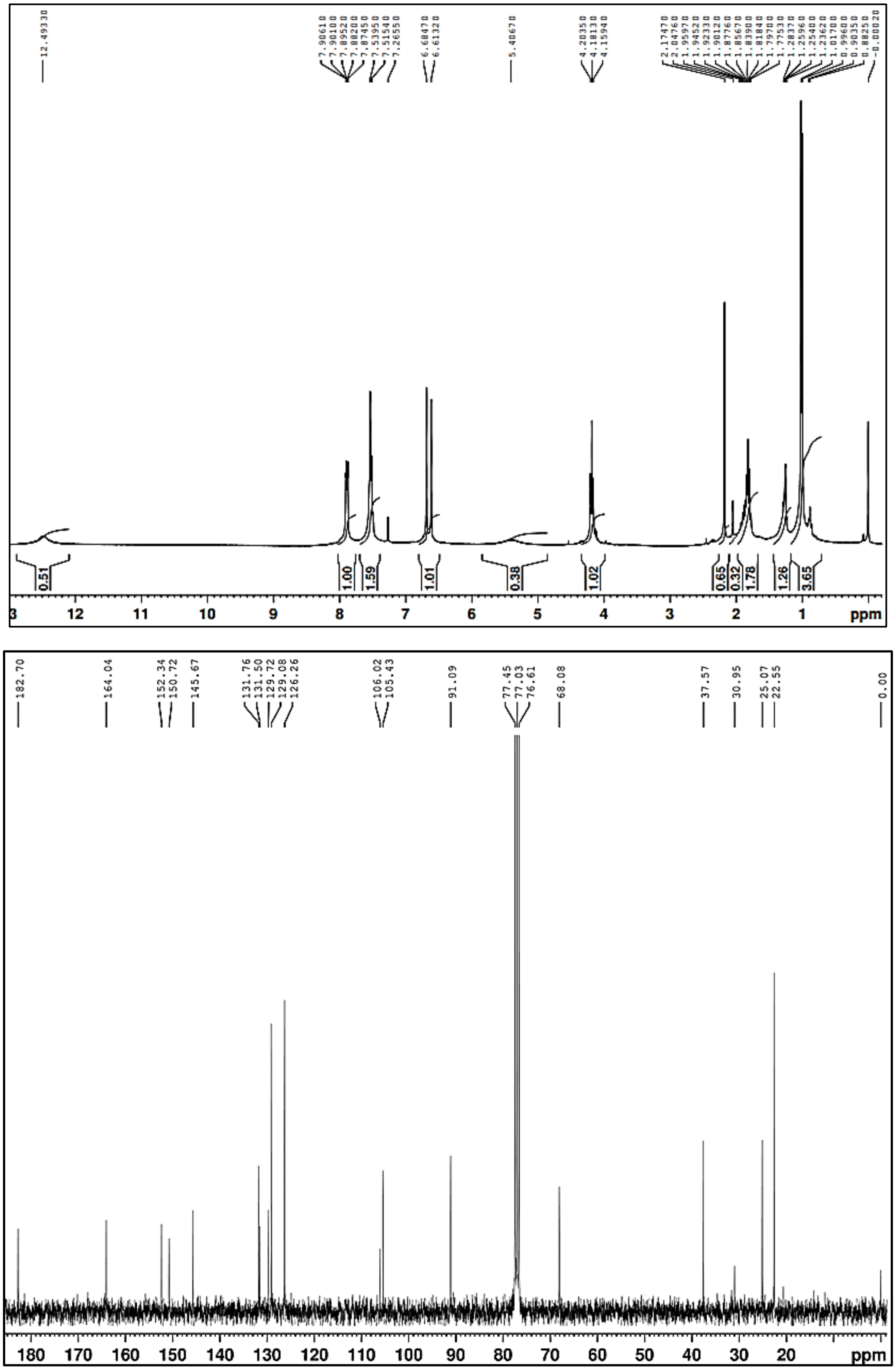

Figure S3. ^{1}H and ^{13}C NMR of compound **13**.

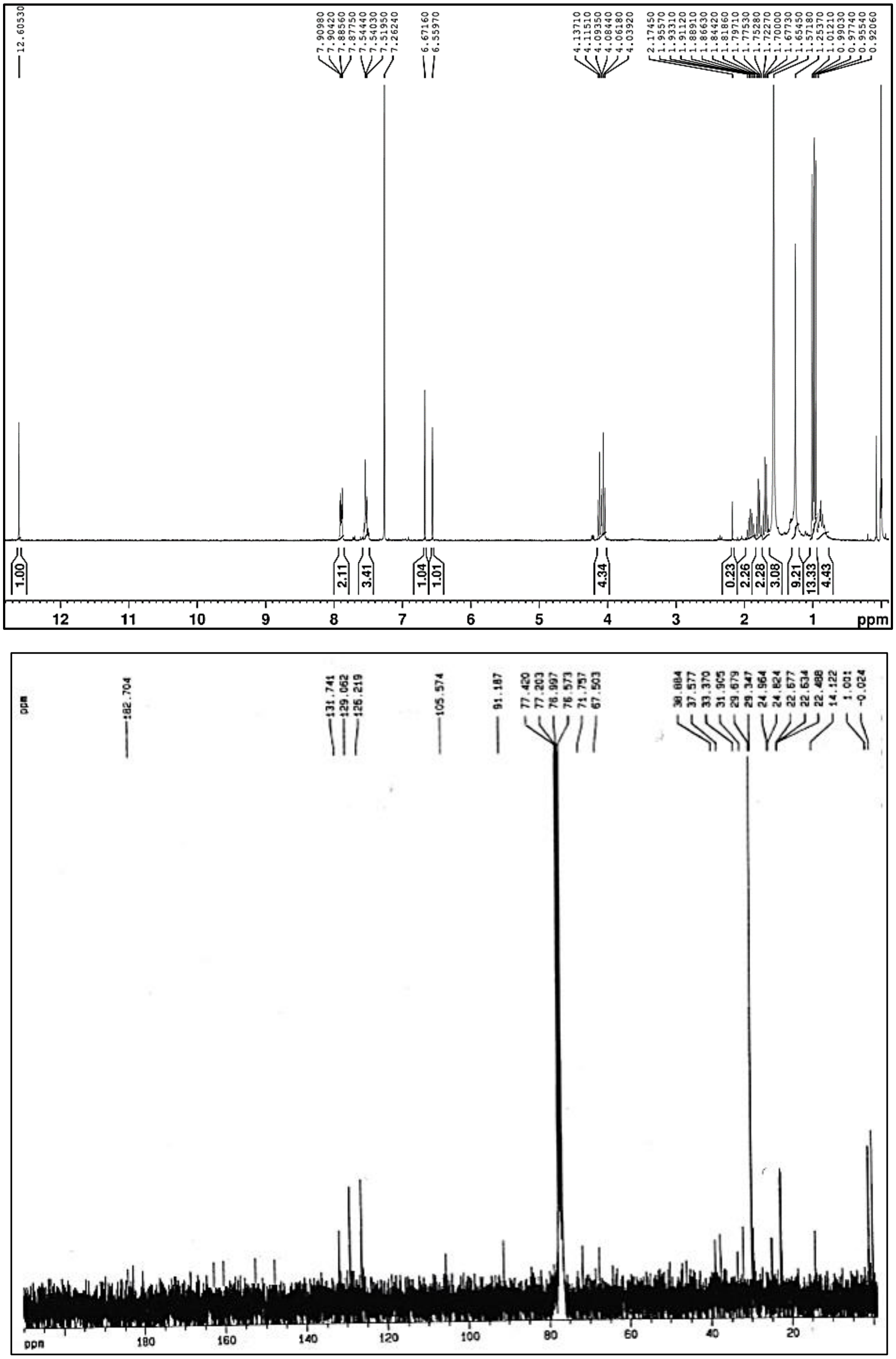

Figure S4. ^{1}H and ^{13}C NMR of compound **14**.

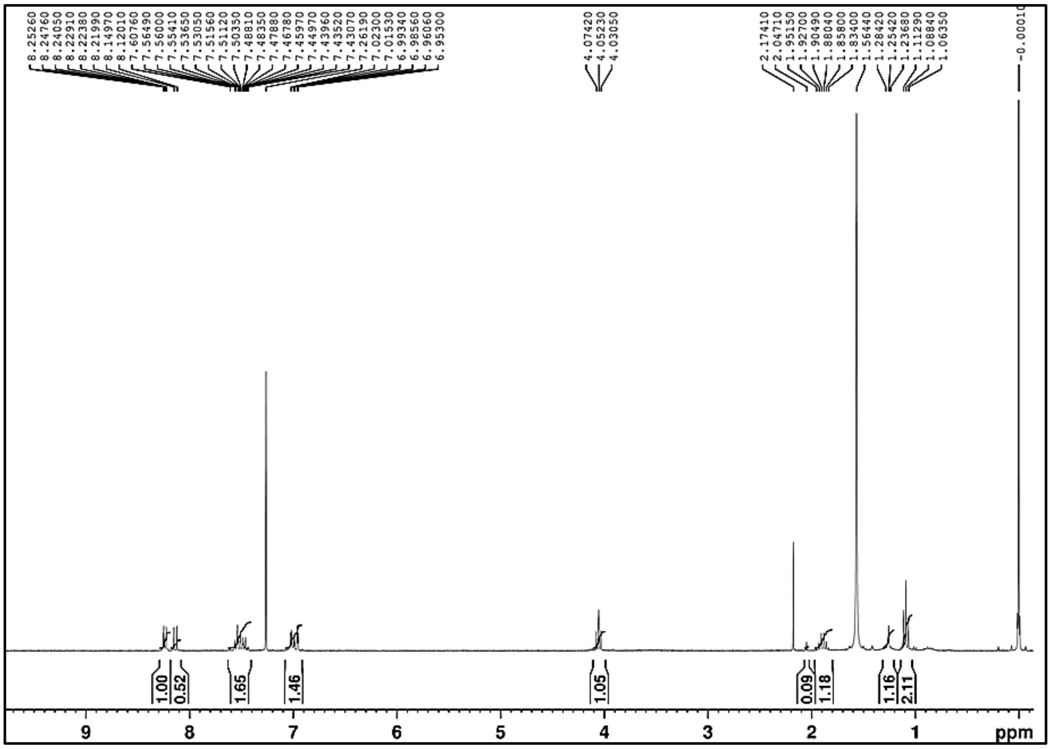

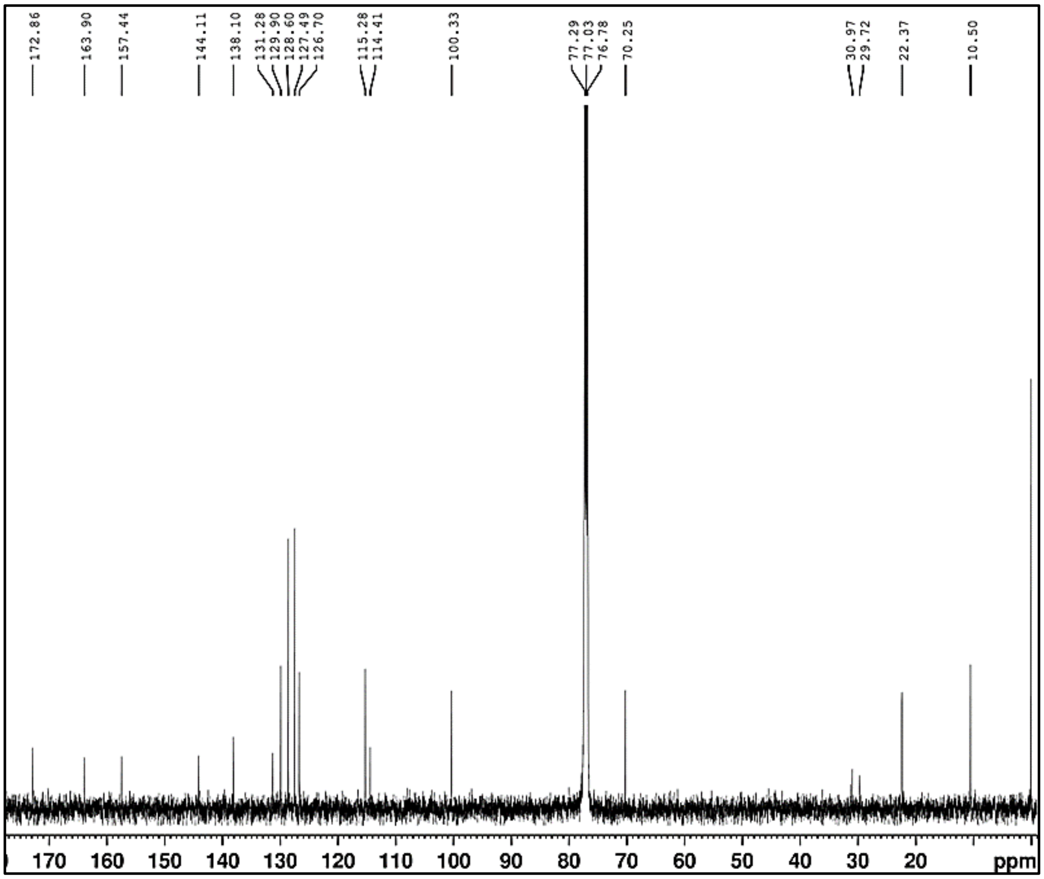

Figure S5. ^{1}H and ^{13}C NMR of compound **17**.

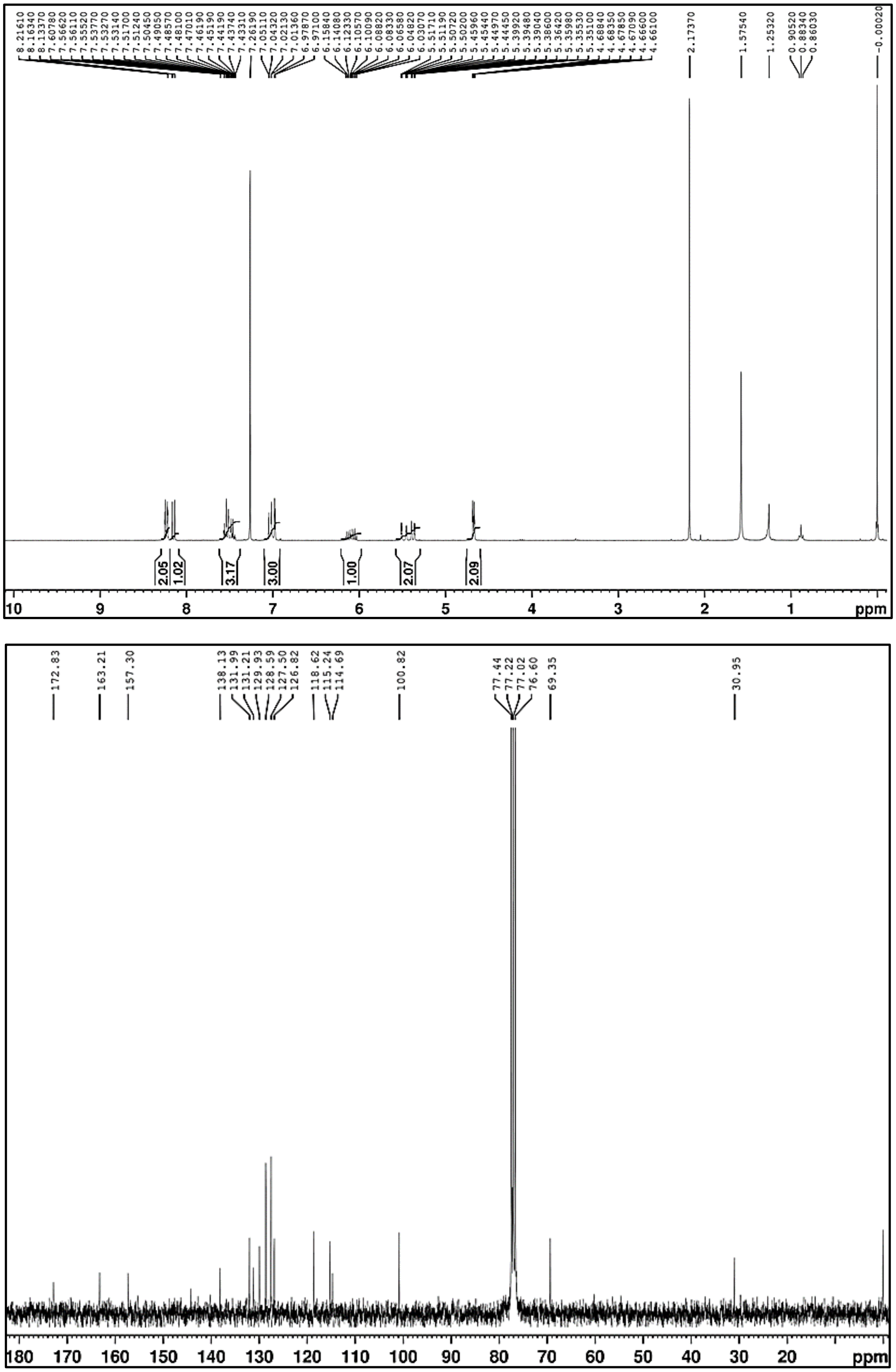

Figure S6. ^{1}H and ^{13}C NMR of compound **18**.

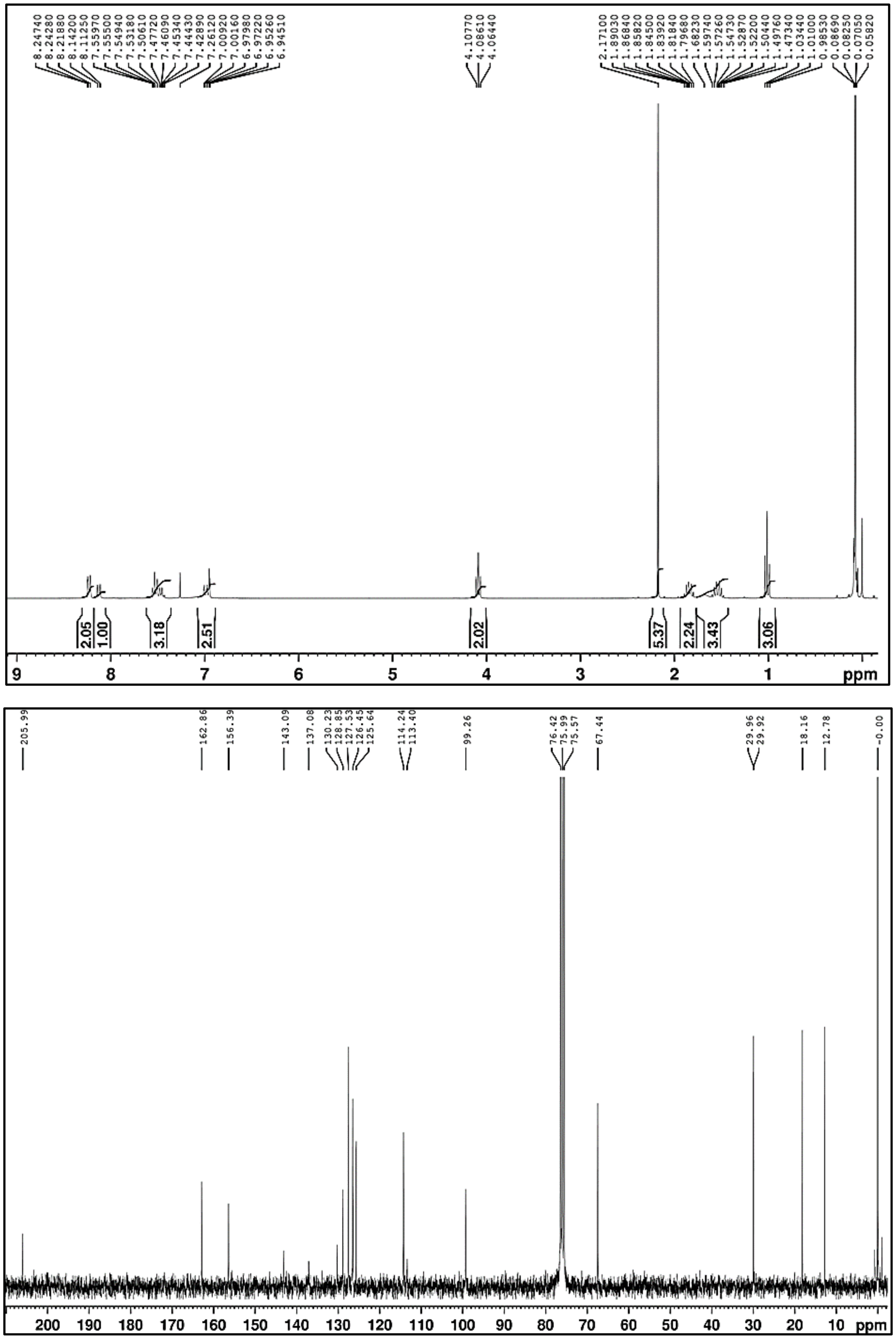

Figure S7. ^{1}H and ^{13}C NMR of compound **19**.

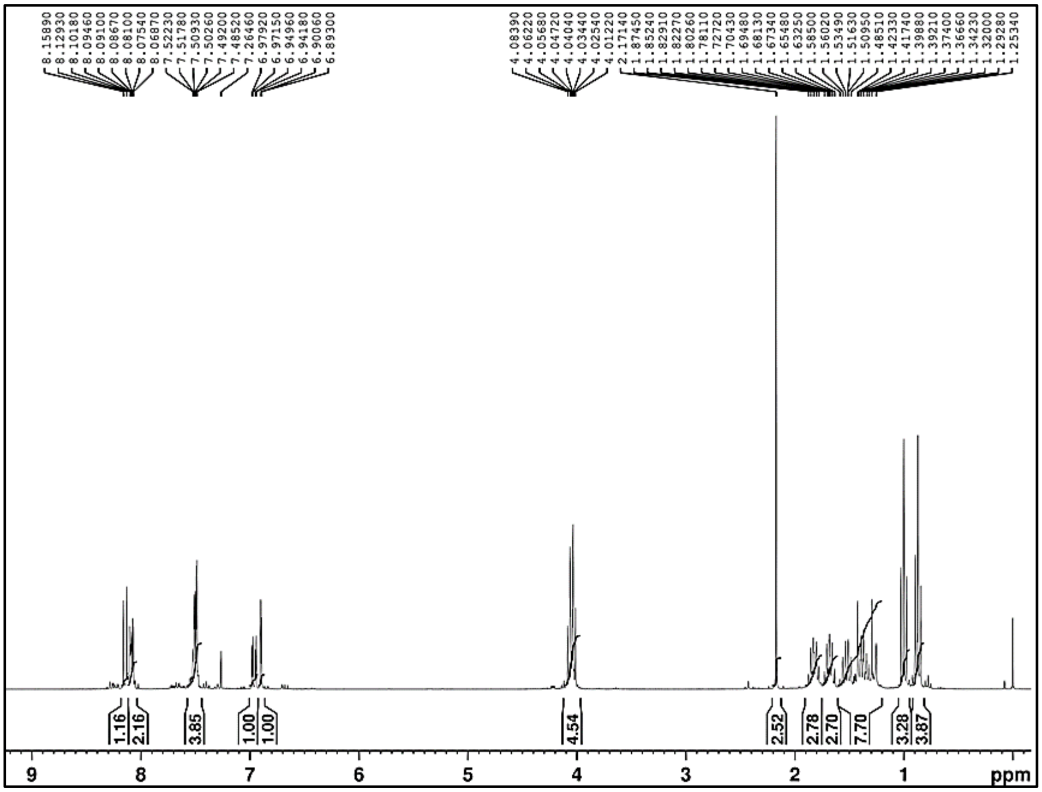

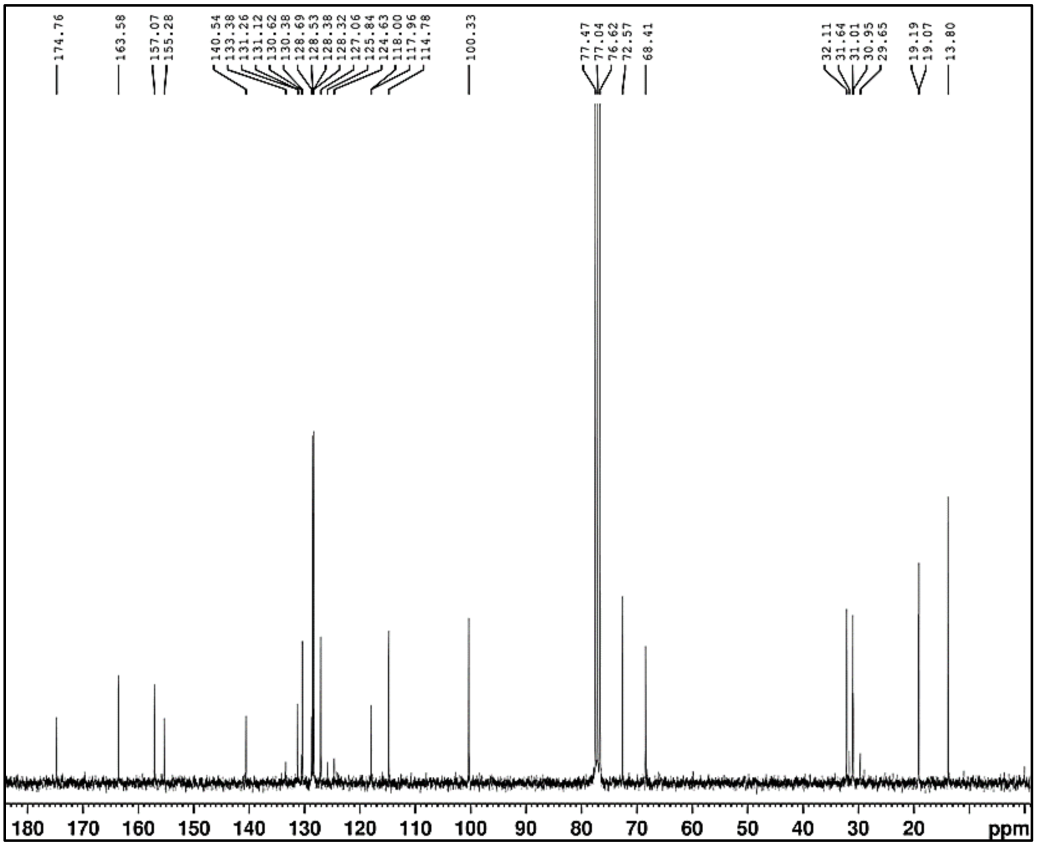

Figure S8. ^{1}H and ^{13}C NMR of compound **20**.

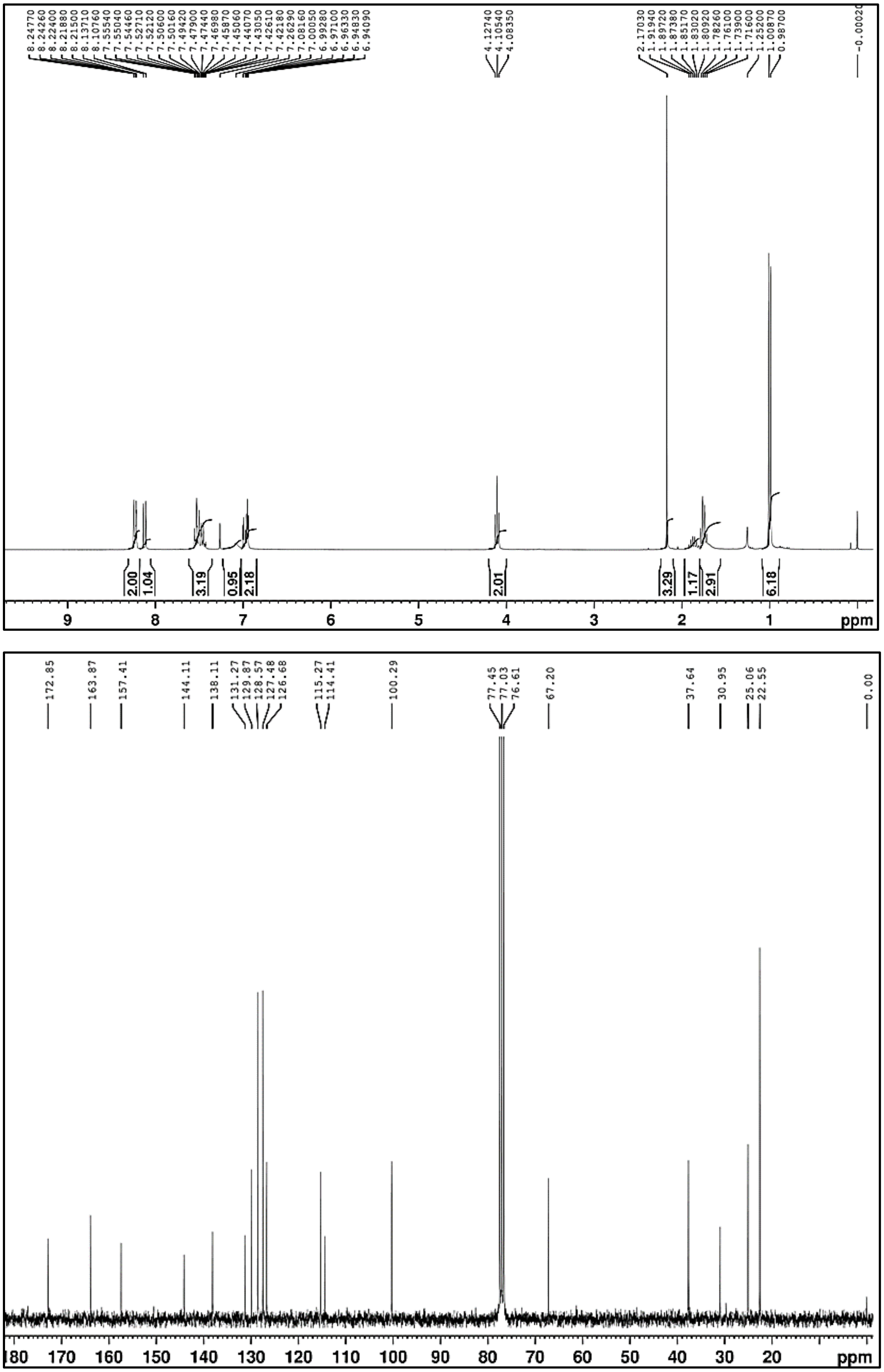

Figure S9. ^{1}H and ^{13}C NMR of compound **21**.

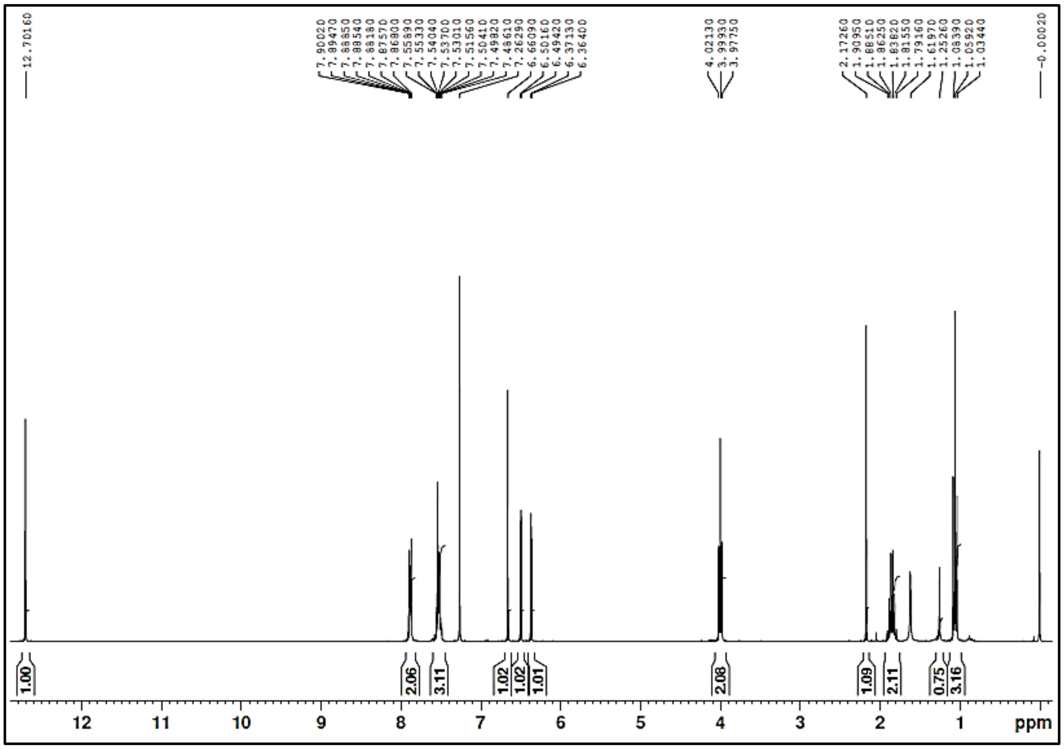

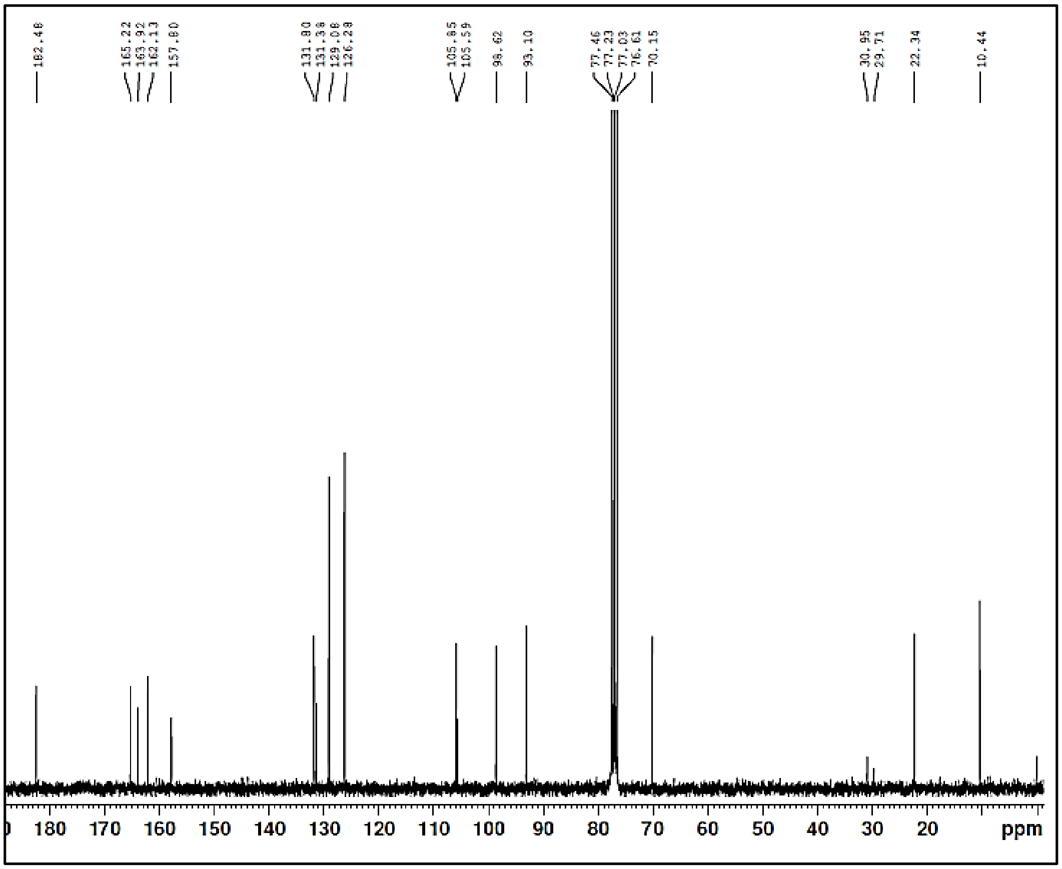

Figure S10. ^{1}H and ^{13}C NMR of compound **24**.

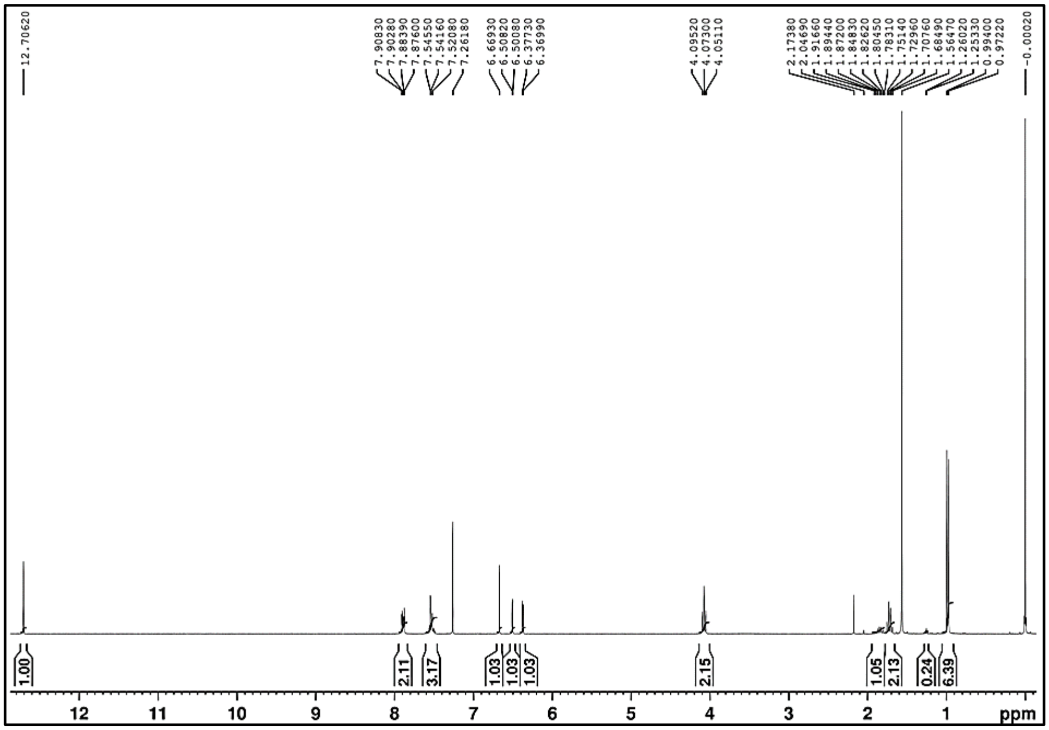

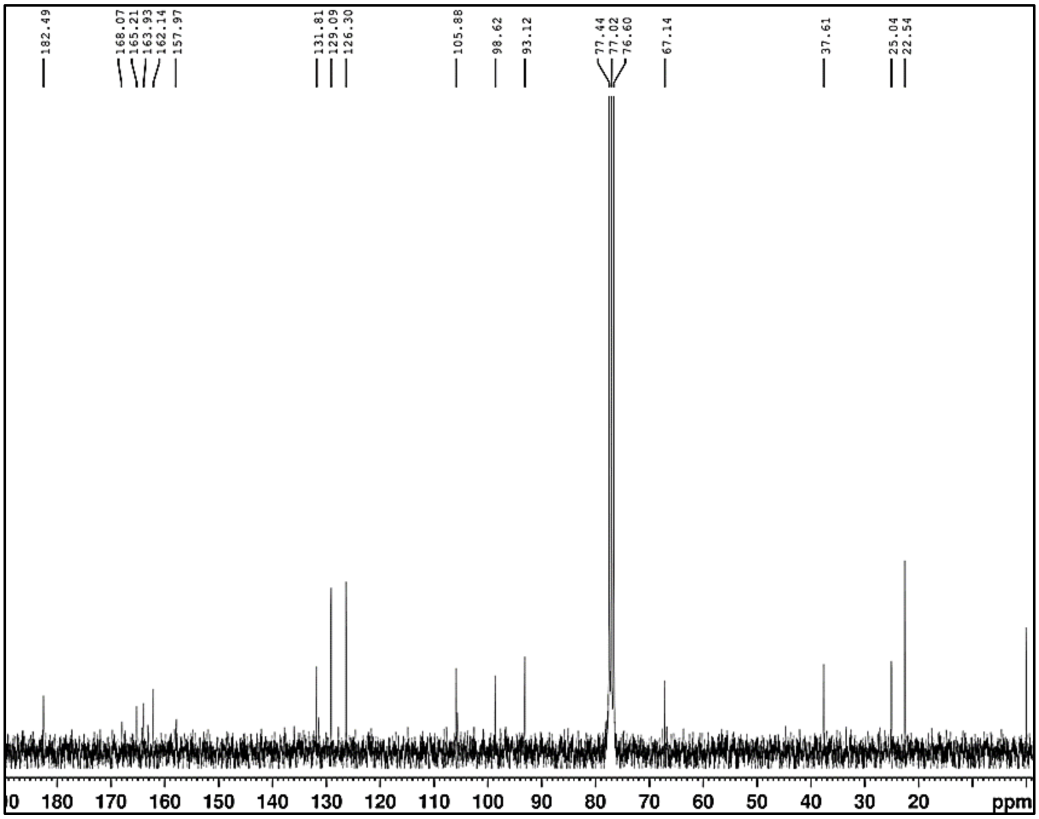

Figure S11. ^{1}H and ^{13}C NMR of compound **27**.

HRMS spectra

Mass Spectrum List Report

Analysis Info	ESI -TOF		
Analysis Name	JRHC1616446000001.d	Acquisition Date	10/18/2016 12:29:37 PM
Sample Name	BETIL 1	Instrument	micrOTOF

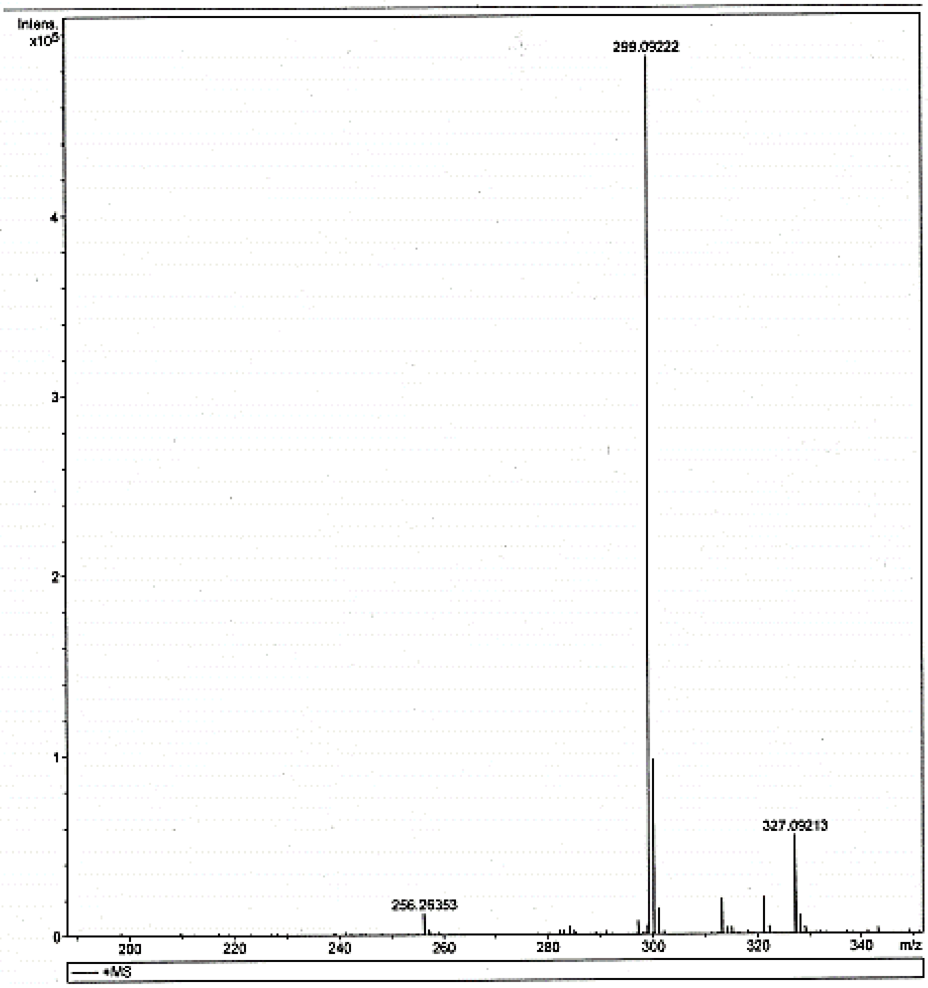

Mass Spectrum Molecular Formula Report

Meas. m/z	#	Formula	Score	m/z	err [mDa]	err [ppm]	mSigma	rdb	e⁻ Conf	N-Rule
299.09222	1	C 17 H 15 O 5	100.00	299.09140	-0.8	-2.7	9.1	10.5	even	ok
321.07421	1	C 17 H 14 Na O 5	100.00	321.07334	-0.9	-2.7	107.2	10.5	even	ok

Figure S12. HRMS of compound **6**.

Mass Spectrum List Report

Analysis Info ESI -TOF
Analysis Name JRHC1616444000001.d
Sample Name BROP 1

Acquisition Date 10/18/2016 12:10:52 PM
Instrument micrOTOF

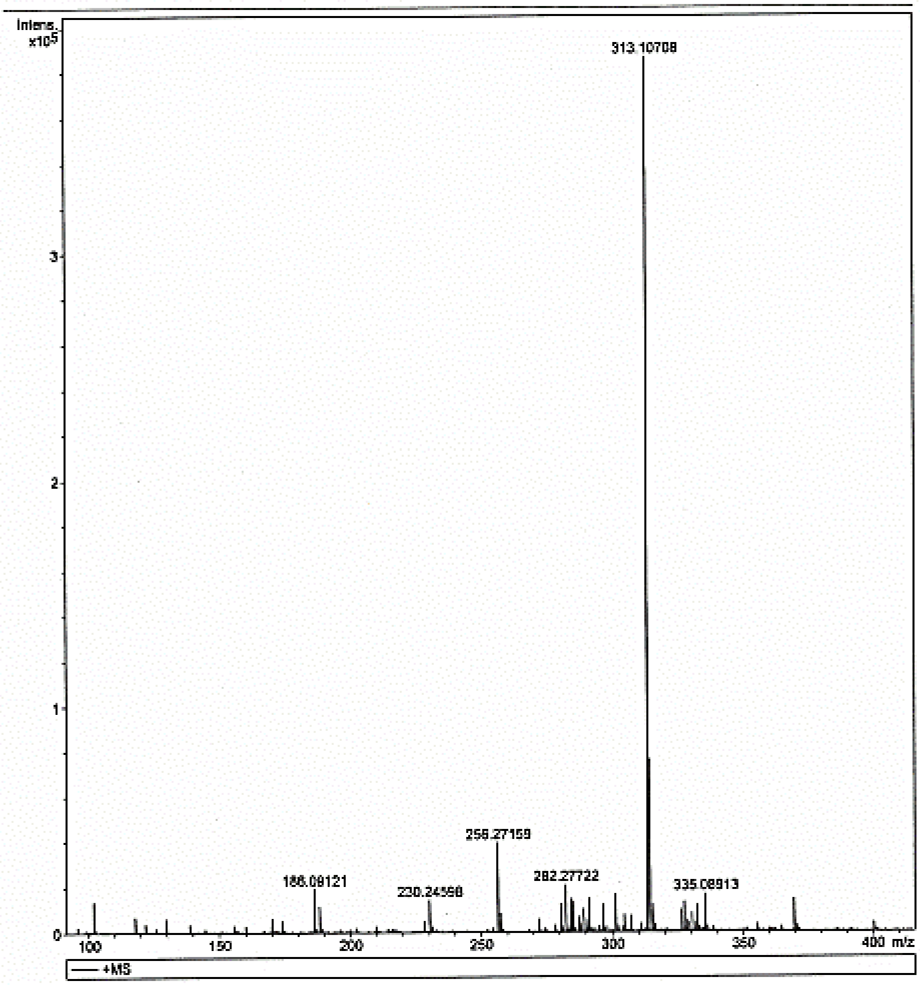

U. Vigo / CACTI / DEPyG

Page 1 of 2

Mass Spectrum Molecular Formula Report

Meas. m/z	#	Formula	Score	m/z	err [mDa]	err [ppm]	mSigma	rdb	e⁻ Conf	N-Rule
313.10708	1	C 18 H 17 O 5	100.00	313.10705	-0.0	-0.1	2.5	10.5	even	ok
335.08913	1	C 18 H 16 Na O 5	100.00	335.08899	-0.1	-0.4	113.6	10.5	even	ok

Figure S13. HRMS of compound **7**.

Mass Spectrum List Report

Analysis Info	ESI -TOF		
Analysis Name	JRHC1616441000001.d	Acquisition Date	10/18/2016 11:45:41 AM
Sample Name	BISO 1	Instrument	micrOTOF

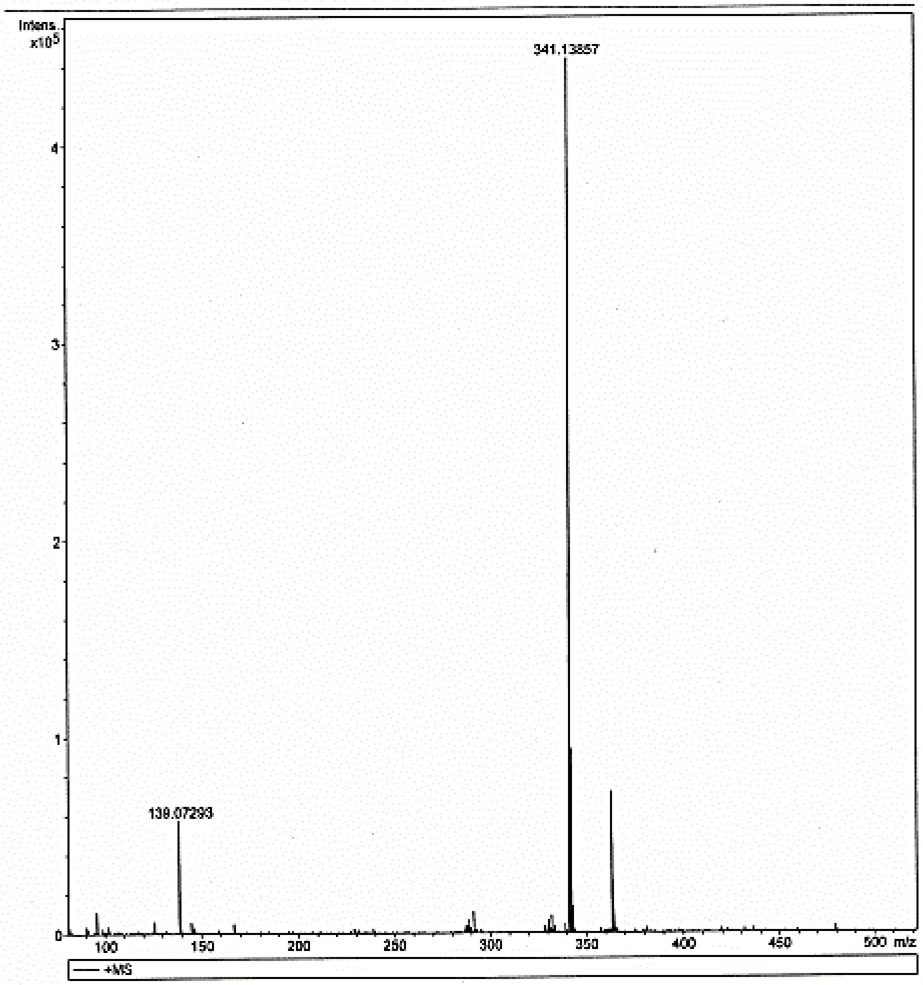

U. Vigo / CACTI / DEPyG

Page 1 of 2

Mass Spectrum Molecular Formula Report

Meas. m/z	#	Formula	Score	m/z	err [mDa]	err [ppm]	mSigma	rdb	e⁻ Conf	N-Rule
341.13857	1	C 20 H 21 O 5	100.00	341.13835	-0.2	-0.6	3.3	10.5	even	ok
363.12027	1	C 20 H 20 Na O 5	100.00	363.12029	0.0	0.1	21.9	10.5	even	ok

Figure S14. HRMS of compound **13**.

Mass Spectrum List Report

Analysis Info	ESI -TOF		
Analysis Name	JRHC1616442000001.d	Acquisition Date	10/18/2016 11:53:31 AM
Sample Name	BISO 2	Instrument	micrOTOF

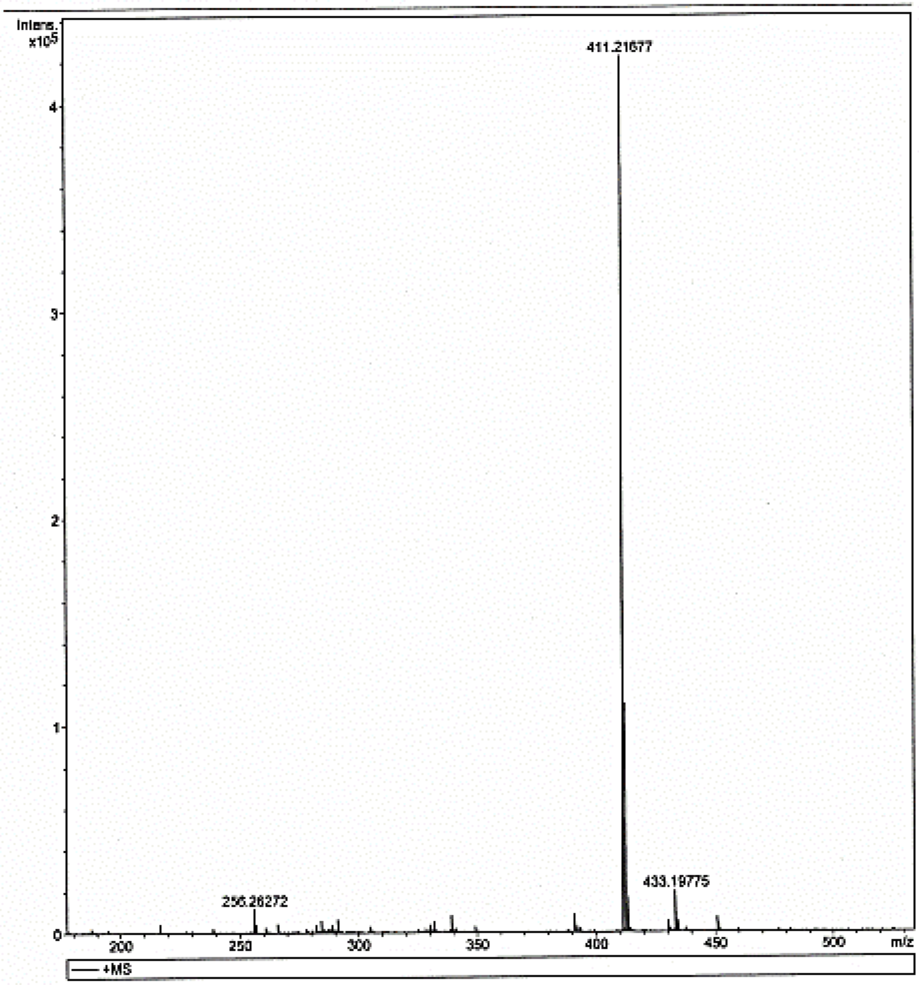

U. Vigo / CACTI / DEPyG

Mass Spectrum Molecular Formula Report

Meas. m/z	#	Formula	Score	m/z	err [mDa]	err [ppm]	mSigma	rdb	e⁻ Conf	N-Rule
411.21677	1	C 25 H 31 O 5	100.00	411.21660	-0.2	-0.4	8.3	10.5	even	ok
433.19775	1	C 25 H 30 Na O 5	100.00	433.19855	0.8	1.8	26.4	10.5	even	ok

Figure S15. HRMS of compound **14**.

Mass Spectrum List Report

Analysis Info ESI -TOF
Analysis Name JRHC15154410000001.d
Sample Name HRDP

Acquisition Date 10/18/2016 1:10:05 PM
Instrument micrOTOF

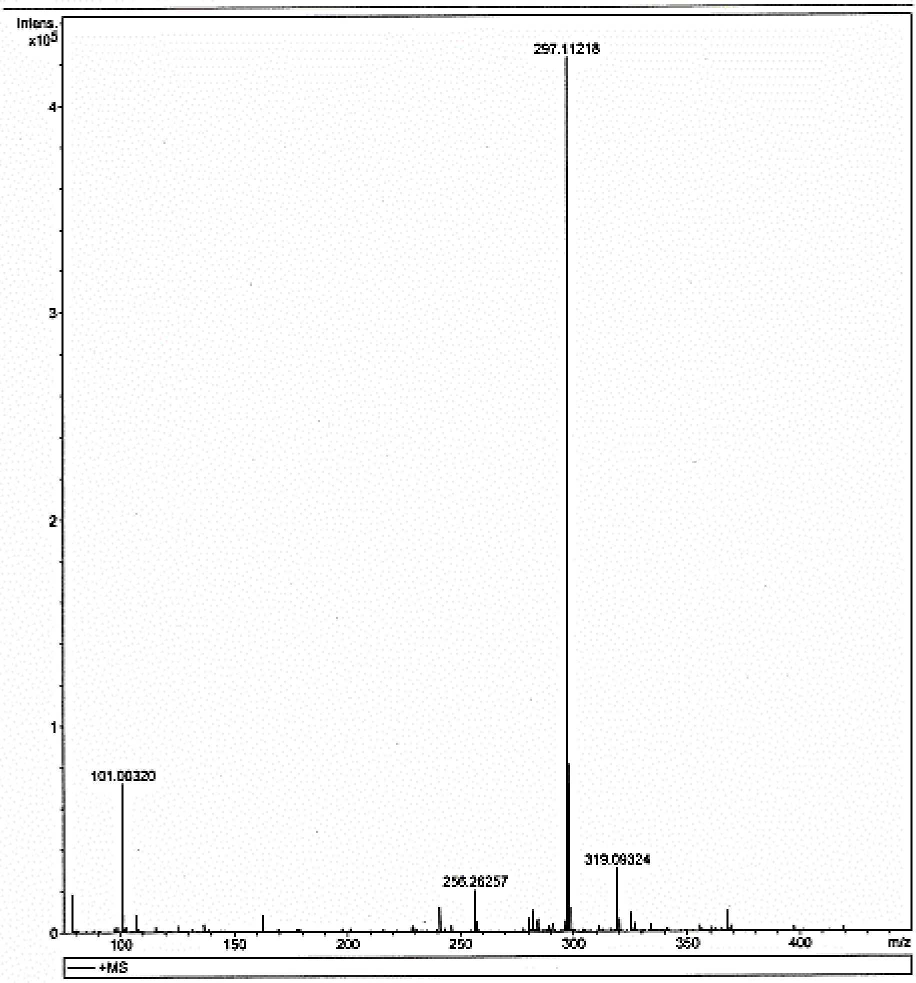

U. Vigo / CACTI / DEPyG

Page 1 of 2

Mass Spectrum Molecular Formula Report

Meas. m/z	#	Formula	Score	m/z	err [mDa]	err [ppm]	mSigma	rdb	e⁻ Conf	N-Rule
297.11218	1	C 18 H 17 O 4	100.00	297.11214	-0.0	-0.2	2.0	10.5	even	ok
319.09324	1	C 18 H 16 Na O 4	100.00	319.09408	0.8	2.6	15.8	10.5	even	ok

Figure S16. HRMS of compound **17**.

Mass Spectrum List Report

Analysis Info ESI -TOF

Analysis Name JRHC16164411000001.d   Acquisition Date 10/18/2016 1:17:57 PM

Sample Name HALI   Instrument micrOTOF

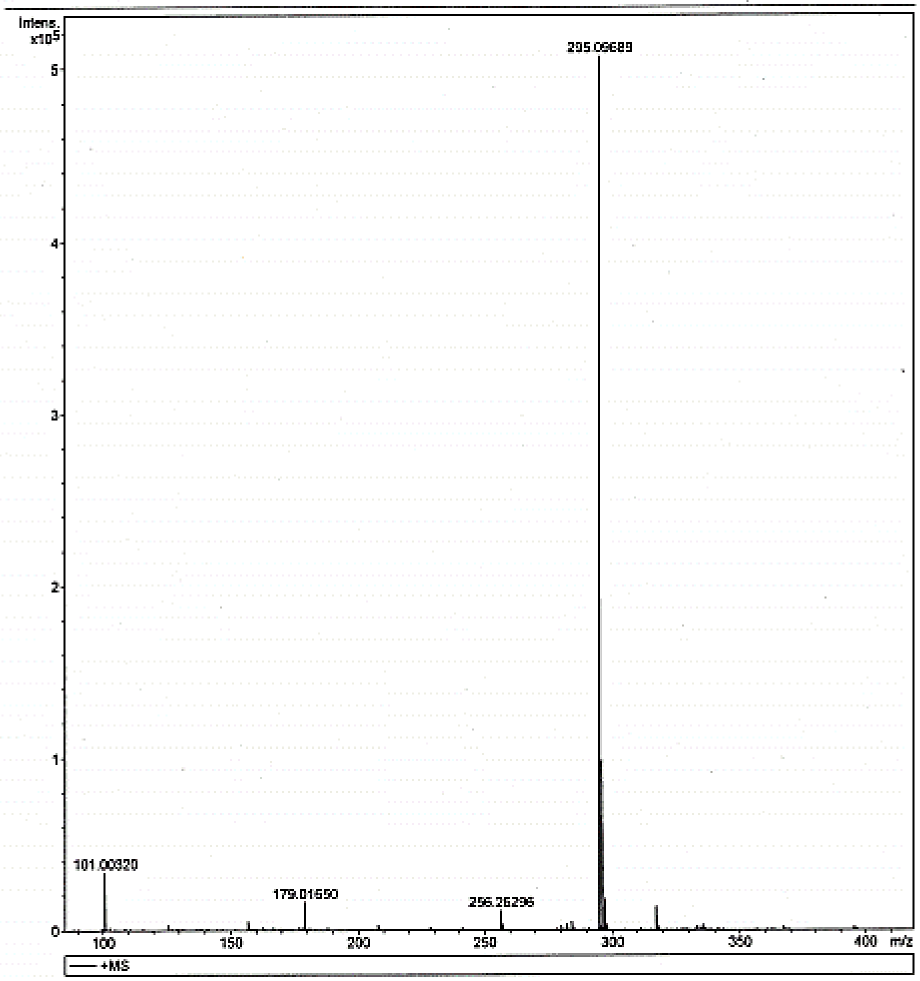

U. Vigo / CACTI / DEPyG   Page 1 of 2

Mass Spectrum Molecular Formula Report

Meas. m/z	#	Formula	Score	m/z	err [mDa]	err [ppm]	mSigma	rdb	e⁻ Conf	N-Rule
295.09689	1	C 18 H 15 O 4	100.00	295.09649	-0.4	-1.4	5.8	11.5	even	ok
317.07857	1	C 18 H 14 Na O 4	100.00	317.07843	-0.2	-0.8	113.4	11.5	even	ok

Figure S17. HRMS of compound **18**.

Mass Spectrum List Report

Analysis Info ESI -TOF

Analysis Name JRHC1616448000001.d

Sample Name HUTIL 1

Acquisition Date 10/19/2016 12:44:36 PM

Instrument micrOTOF

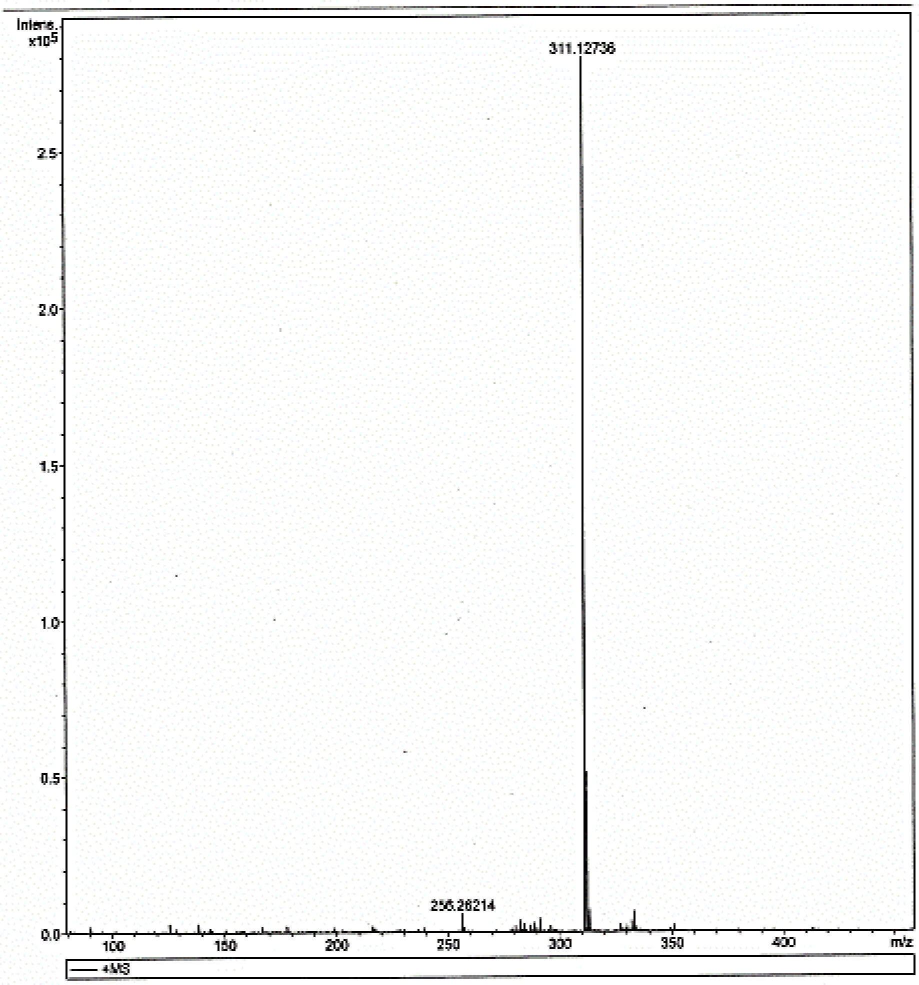

U. Vigo / CACTI / DEPyG

Page 1 of 2

Mass Spectrum Molecular Formula Report

Meas. m/z	#	Formula	Score	m/z	err [mDa]	err [ppm]	mSigma	rdb	e⁻ Conf	N-Rule
311.12736	1	C 19 H 19 O 4	100.00	311.12779	0.4	1.4	13.0	10.5	even	ok
333.10993	1	C 19 H 18 Na O 4	100.00	333.10973	-0.2	-0.6	119.8	10.5	even	ok

Figure S18. HRMS of compound **19**.

Mass Spectrum List Report

**Analysis Info**	ESI -TOF		
Analysis Name	JRHC1616449000001.d	Acquisition Date	10/18/2016 12:52:20 PM
Sample Name	HUTIL 2	Instrument	micrOTOF

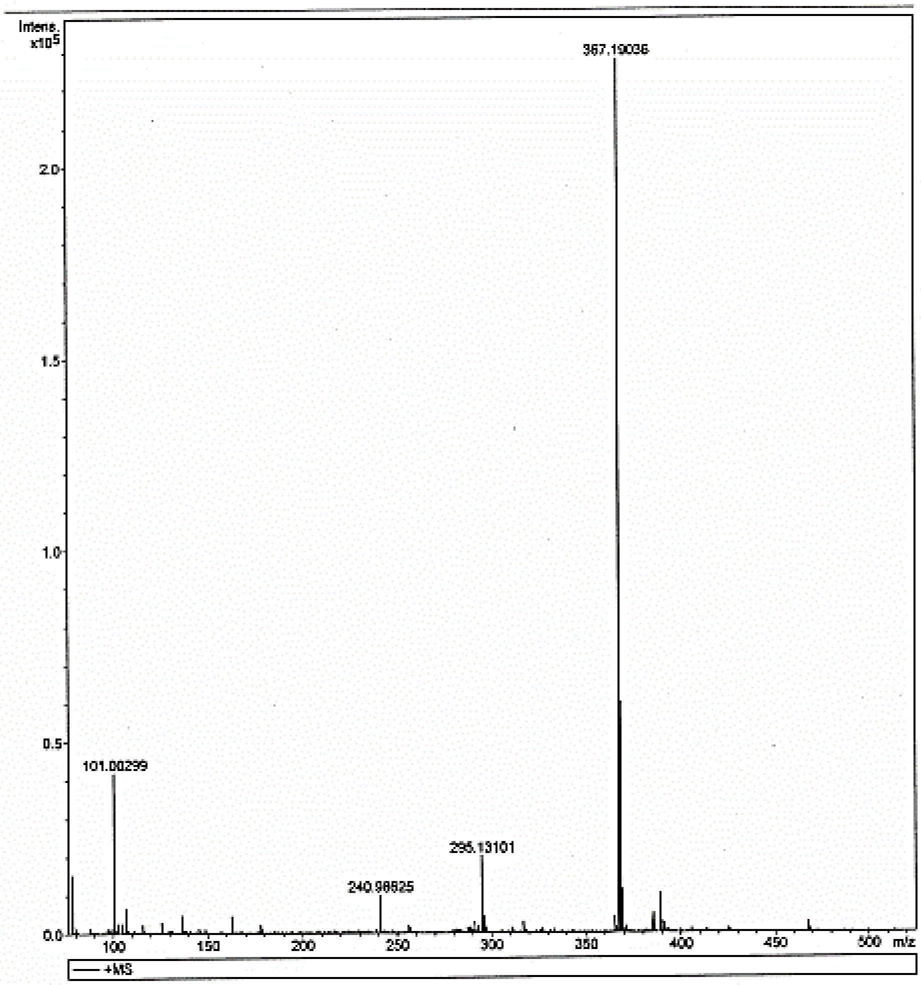

U. Vigo / CACTI / DEPyG

Page 1 of 2

Mass Spectrum Molecular Formula Report

Meas. m/z	#	Formula	Score	m/z	err [mDa]	err [ppm]	mSigma	rdb	e⁻ Conf	N-Rule
367.19036	1	C 23 H 27 O 4	100.00	367.19039	0.0	0.1	11.3	10.5	even	ok
389.17123	1	C 23 H 26 Na O 4	100.00	389.17233	1.1	2.8	28.8	10.5	even	ok

Figure S19. HRMS of compound **20**.

Mass Spectrum List Report

**Analysis Info**	**ESI -TOF**		
**Analysis Name**	**JRHC1616447000001.d**	**Acquisition Date**	**10/18/2016 12:37:54 PM**
**Sample Name**	**HISO**	**Instrument**	**micrOTOF**

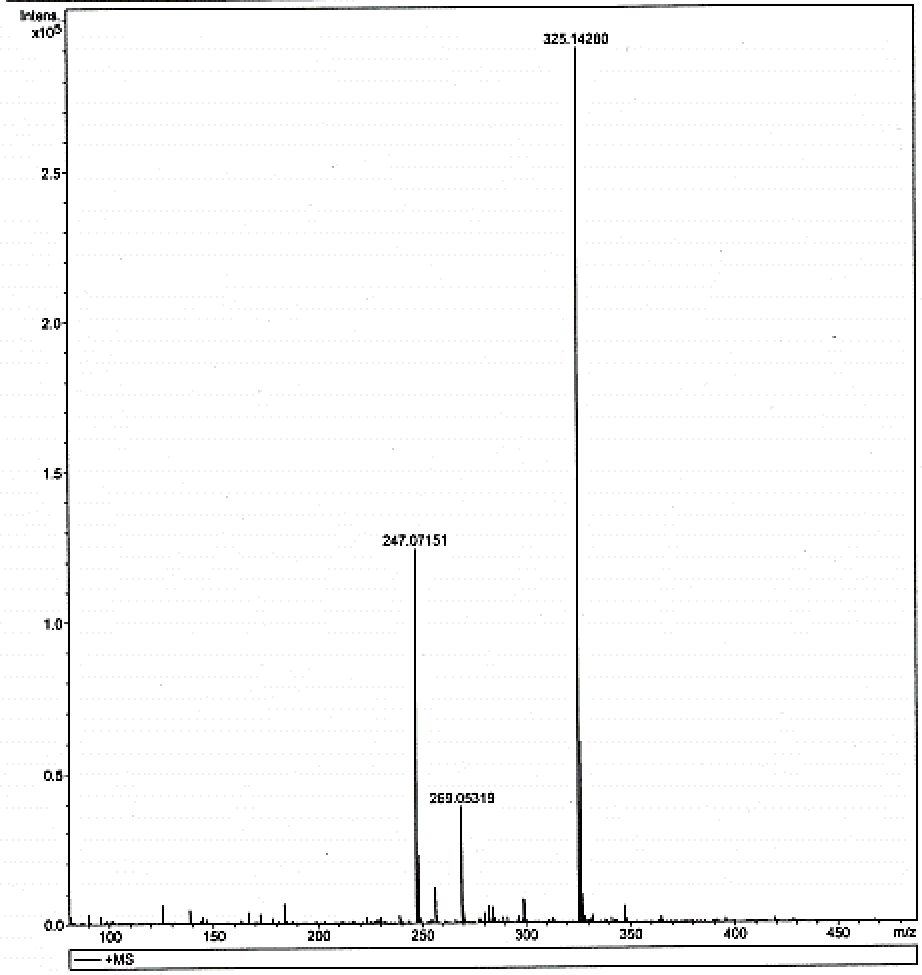

U. Vigo / CACTI / DEPyG **Page 1 of 2**

Mass Spectrum Molecular Formula Report

Meas. m/z	#	Formula	Score	m/z	err [mDa]	err [ppm]	mSigma	rdb	e⁻ Conf	N-Rule
325.14200	1	C 20 H 21 O 4	100.00	325.14344	0.6	2.0	5.1	10.5	even	ok

Figure S20. HRMS of compound **21**.

Mass Spectrum List Report

**Analysis Info**	**ESI -TOF**		
Analysis Name	JRHC16164416000001.d	Acquisition Date	10/18/2016 1:49:55 PM
Sample Name	**CROP**	Instrument	micrOTOF

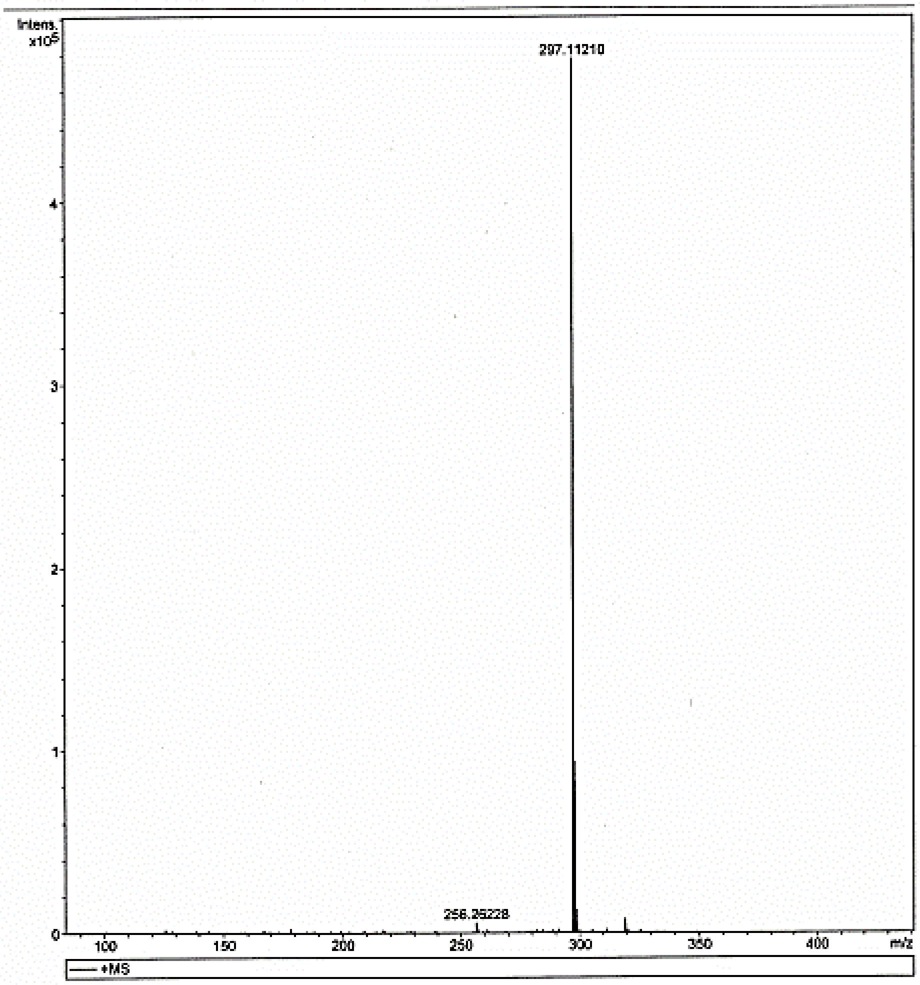

U. Vigo / CACTI / DEPyG Page 1 of 2

Mass Spectrum Molecular Formula Report

Meas. m/z	#	Formula	Score	m/z	err [mDa]	err [ppm]	mSigma	rdb	e⁻ Conf	N-Rule
297.11210	1	C 18 H 17 O 4	100.00	297.11214	0.0	0.1	0.6	10.5	even	ok

Figure S21. HRMS of compound **24**.

Mass Spectrum List Report

Analysis Info	ESI -TOF		
Analysis Name	JRHC15164414000002.d	Acquisition Date	10/18/2016 1:37:37 PM
Sample Name	CISO	Instrument	micrOTOF

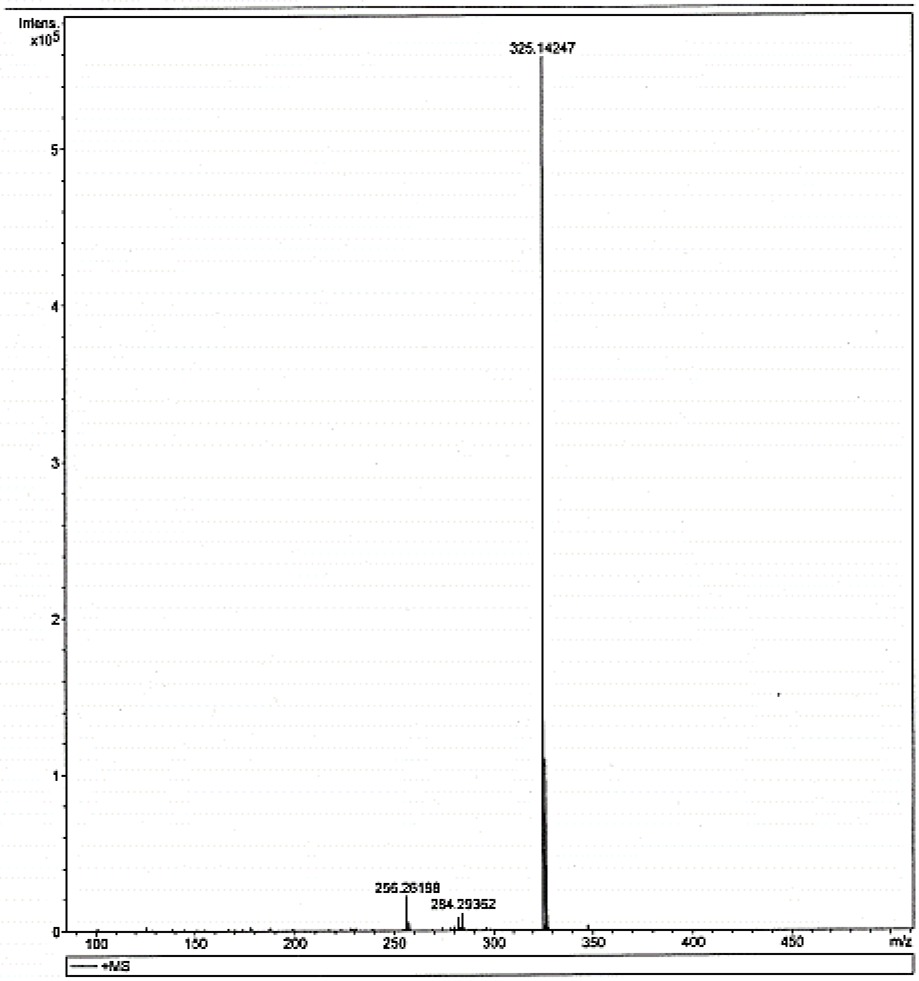

U. Vigo / CACTI / DEPyG

Page 1 of 2

Mass Spectrum Molecular Formula Report

Meas. m/z	#	Formula	Score	m/z	err [mDa]	err [ppm]	mSigma	rdb	e⁻ Conf	N-Rule
325.14247	1	C 20 H 21 O 4	100.00	325.14344	1.0	3.0	11.2	10.5	even	ok

Figure S22. HRMS of compound **27**.
